# Satellite data show trees delay budburst across landscapes to escape herbivores

**DOI:** 10.1038/s41559-026-03071-9

**Published:** 2026-05-01

**Authors:** Soumen Mallick, Jens Lichter, Soyeon Bae, Thomas Kneib, Freerk Molleman, Benjamin M. L. Leroy, Torben Hilmers, Maike Huszarik, Andrew M. Liebhold, Wolfgang W. Weisser, Johannes A. Jehle, Jörg Müller, Andreas Prinzing

**Affiliations:** 1 Ecological Station Fabrikschleichach, Biocenter, University of Würzburg, Rauhenebrach, Germany; 2 Statistics and Campus Institute Data Science, University of Göttingen, Goettingen, Germany; 3 Centre of Biodiversity and Sustainable Land Use, University of Göttingen, Goettingen, Germany; 4 Thünen Earth Observation (ThEO), Thünen Institute of Farm Economics, Braunschweig, Germany; 5 Department of Systematic Zoology, Institute of Environmental Biology, Faculty of Biology, A. Mickiewicz University Poznań, Poznań, Poland; 6 Terrestrial Ecology Research Group, Department of Life Science Systems, School of Life Sciences, Technical University of Munich, Freising, Germany; 7 Laboratory Agronomy and Environment, University of Lorraine, INRAE, Colmar, France; 8 Tree Growth and Wood Physiology, TUM School of Life Sciences, Technical University of Munich, Freising, Germany; 9 Faculty of Forestry and Wood Sciences, Czech University of Life Sciences Prague, Prague, Czech Republic; 10 Institute for Biological Control, Julius Kühn Institute, Dossenheim, Germany; 11 Bavarian Forest National Park, Grafenau, Germany; 12 Centre National de la Recherche Scientifique, Research Unit UMR 6553, Ecosystèmes Biodiversité Evolution (ECOBIO), University of Rennes, Rennes, France

**Keywords:** Phenology, Forest ecology, Plant ecology

## Abstract

In recent years, budburst, the timing of leaf emergence, has advanced less than expected despite continued spring warming, suggesting counteracting ecological forces. One of these forces might be increased and earlier herbivory on young leaves under climate warming. Here using 5 years of satellite radar data from 27,500 pixels (10 ×10 m^2^) across 60 temperate oak forest sites under experimental manipulation of insect herbivore loads in Central Europe, we show that prior-year leaf herbivory delayed budburst by 3 days, cancelling the phenological advance observed during a decade of warming. This delay reduced subsequent herbivory by 55%, exceeding the effects of parasitoids or pathogens, and persisted even during pest outbreaks. Across landscapes, the delay was strongest where it probably provided the highest benefit, that is, where a given amount of delay most effectively reduced following herbivory, which suggests an adaptive tree defence. Ultimately, trees may be trapped between responding to two opposing consequences of global change: warming selects for earlier budburst, whereas herbivory selects for delay. Our results underscore the need to consider not only climate, but also plant–herbivore interactions and adaptive evolution to predict tree responses to a changing world.

## Abstract (web summary)

Plant phenology tends to track with climate warming, but it could also be sensitive to biotic factors. This study from a temperate forest landscape in Germany shows that a single year of tree responses to intense insect herbivory can counteract a decade of environmentally driven shifts in spring phenology.

## Main

Plant phenology has been studied in relation to numerous factors. Photoperiod^1,2^, for instance, has been shown to prevent premature budburst (leaf emergence). In addition, the nutritional status of trees^3^, particularly internal nutrient reserves accumulated in previous seasons^4^, can influence phenological development of plants by affecting growth capacity and metabolic readiness. Plant phenology has probably been most extensively studied in relation to climate change^5–12^, with one of the best-known examples being the earlier budburst of trees under elevated spring temperatures^6–10^. However, the advance in budburst under climate change has been slower than expected^12–14^. Budburst can also be driven by another aspect of climate change: increasing biotic pressures, notably insect herbivores^11,15–17^. Small-scale studies (on ~20 individual trees^18,19^) have suggested that intense leaf herbivory on a tree in one-year delays budburst in the following year. However, cases of advanced budburst after intense leaf herbivory have also been documented^15,20^. It remains unclear which of these patterns on individual trees scales up to entire landscapes, where budburst advancement has unexpectedly slowed^12–14^. The expected budburst advancement under elevated spring temperatures could be suppressed by the herbivory-induced budburst delay. Our hypothesis 1 is thus that, even across entire landscapes, intense leaf herbivory in one year delays budburst in the following year.

**Fig. 1:**
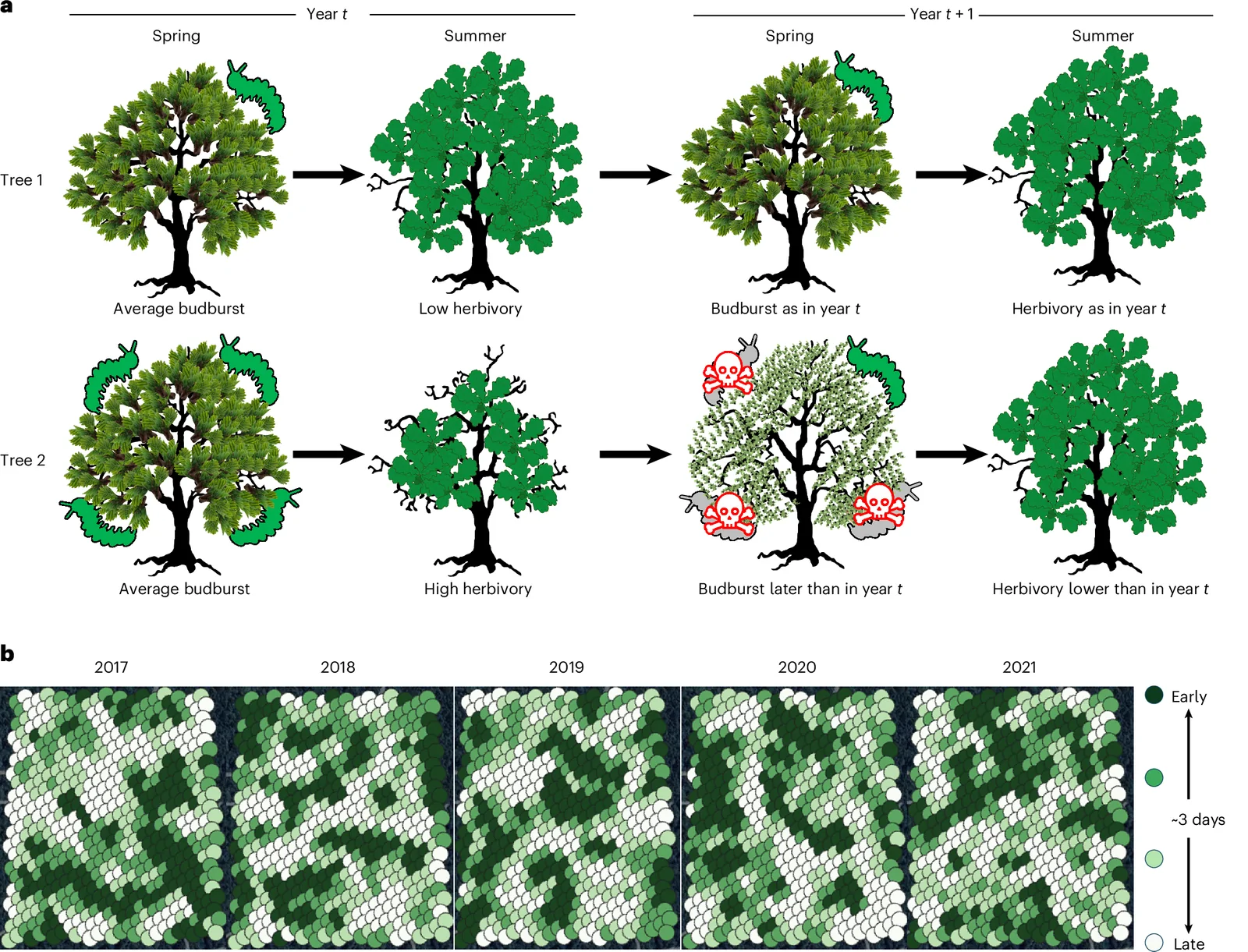
The effect of leaf herbivory by insects on tree budburst and subsequent leaf herbivory. **a**, Conceptual framework of our study. Low herbivory in year *t* (tree 1) does not induce a change in budburst timing in year *t* + 1, resulting in no between-year change in leaf–caterpillar synchrony (living caterpillars) and herbivory. In contrast, high herbivory in year *t* (tree 2) induces a delay in budburst in year *t* + 1, which disrupts synchrony between herbivores and leaf emergence and reduces herbivory by premature caterpillar death (indicated by skulls). **b**, Landscape-scale mosaic of budburst delay in our study area. Each picture shows, for a given year, satellite-detected spatial variability in budburst phenology across pixels (each approximating an individual crown) at one of our 60 sites (site AHC). The extensive interannual variation in budburst timing observed for individual pixels aligns with the variability proposed in the conceptual framework in **a**. Supplementary Video 1 shows three additional sites from the same experimental block (block A, Extended Data Fig. 1).

In temperate forests, the abundance of herbivorous insects typically peaks shortly after budburst, when young leaves are available and highly nutritious^17,21,22^. A substantial portion of the leaf-feeding insect community, often 60–80%, emerge as caterpillars during this period, making spring the peak time for herbivory^22–24^. Therefore, a delay in budburst would lead to spring herbivores emerging before leaves are available (Fig. 1a), enabling trees to escape peak leaf herbivory by disrupting synchrony with their attackers (hypothesis 2). This would result in herbivore mortality and a reduced overall herbivory^16,21^—a form of enemy escape in time that is rarely considered^16,25^. However, the evidence for such a mechanism is lacking.

Existing studies have covered only a narrow range of herbivory intensities^15,18–20^, leaving unresolved the question of whether herbivory-induced budburst delay remains effective during pest outbreaks, when trees are under the greatest pressure to defend themselves. Because outbreak severity is driven largely by transient surges of herbivore populations across entire landscapes rather than by consistent or tree-specific factors^25–28^, herbivory in an outbreak year is likely to be a poor predictor of herbivory risk for individual trees in subsequent years. Under these conditions, we expect that the herbivory-induced budburst delay may be lower at outbreak sites than at non-outbreak sites (hypothesis 3). This effect of outbreak may be particularly seen if trees perceive outbreaks through increased light penetration^29^ or through chemically mediated signals exchanged among conspecifics^30^, mechanisms that enable trees to detect elevated herbivore pressure beyond their own direct experience. Moreover, the common assumption is that severe herbivore outbreaks overwhelm tree defences^26–28,31^, such that even trees that delay budburst may still experience severe defoliation under extreme herbivore densities^21,31,32^. High herbivore densities also increase the likelihood of spillover from neighbouring trees^33^, further reducing the effectiveness of delayed budburst. Consequently, we expect that for a given magnitude of budburst delay, the resulting reduction in herbivory may be lower at outbreak sites than at non-outbreak sites (hypothesis 4).

Finally, because existing studies have covered only limited spatial and temporal scales^15,18–20^, it is still unclear whether herbivory-induced budburst delay represents an adaptive response. Such a delay would be adaptive only if its benefits are high^34–36^. The fitness benefits for trees probably increase with the effectiveness of budburst delay in reducing herbivory. Consequently, if budburst delay functions as a selected, adaptive defence, its magnitude should be greatest in forests where delaying budburst most reliably increases fitness by lessening herbivore damage (hypothesis 5). Detecting such adaptive coupling requires a landscape-scale perspective that captures broad spatiotemporal variability in both budburst and herbivory across sites and years (Fig. 1b).

## Use of satellite-based indices to monitor landscape-scale variability in budburst and herbivory

**Extended Data Fig. 1:**
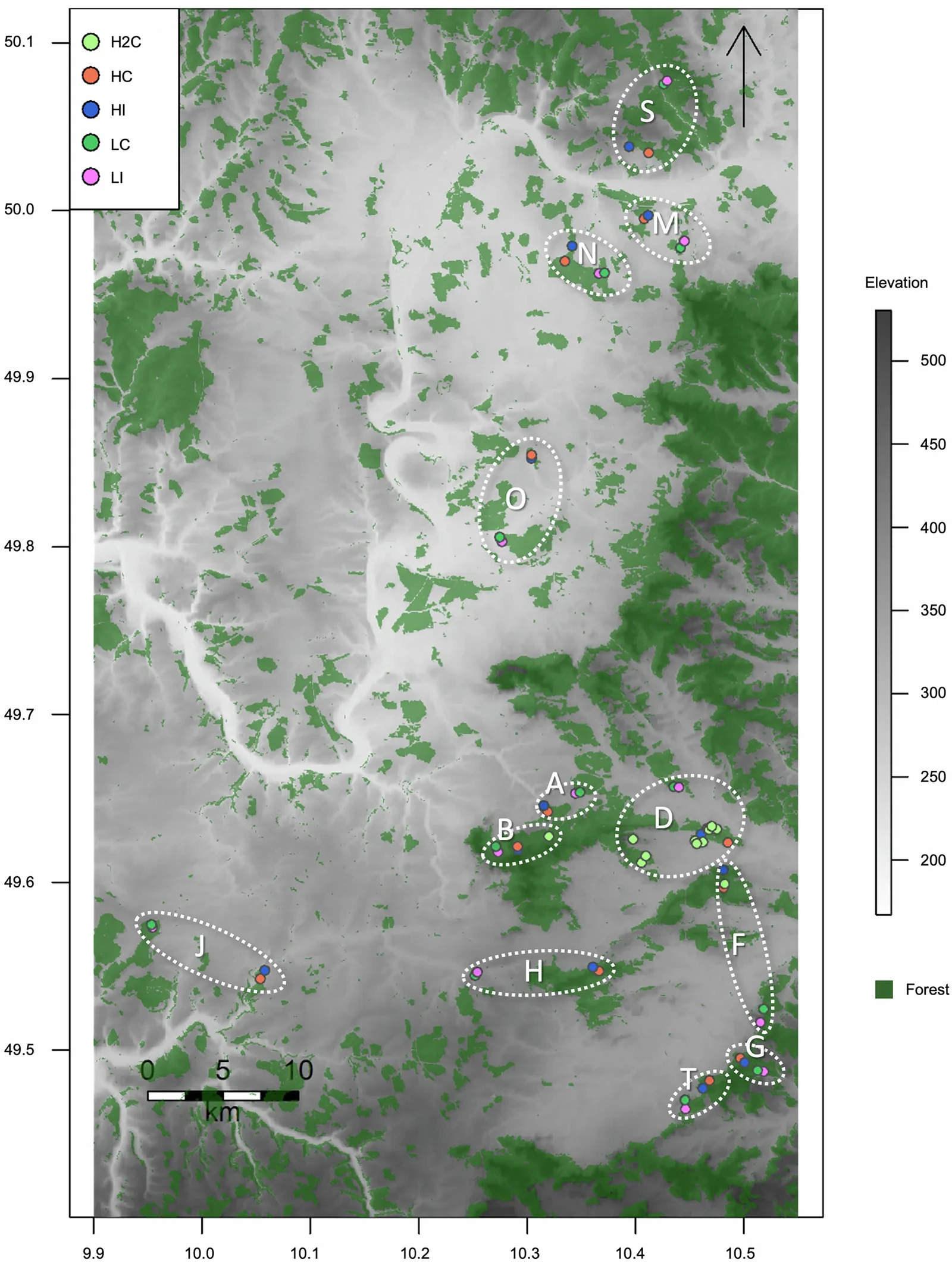
Map of the study area. The map shows 60 forest sites distributed across 12 experimental blocks. Experimental blocks are indicated by ellipses labelled with capital letters (A, B, D, F, G, H, J, M, N, O, S, T). Within each block, a minimum of four sites were established, differing in experimental herbivory treatments. The four treatment combinations (see Methods for details) comprised high defoliation risk unsprayed control sites (HC), high defoliation risk insecticide-treated sites (HI), low defoliation risk unsprayed control sites (LC), and low defoliation risk insecticide-treated sites (LI). In addition, blocks B, D, and F contain extra HC sites, here denoted as H2C.

Monitoring landscape-scale variability in budburst and herbivory requires several observations per week, for several weeks, which is not feasible across thousands of trees in a landscape, let alone across dozens of landscapes. In this study, we overcome these limitations using newly developed satellite-based (Sentinel-1) indices of canopy development^37^, which enable cloud-independent monitoring of budburst and leaf herbivory (Methods). The day-by-day trajectory of this index has been validated against both optical and terrestrial laser scanning data^37^. These indices reliably capture canopy phenology and defoliation at the 10 × 10 m^2^ pixel scale, where each pixel approximately represents the size of an average tree crown. We analysed 27,500 pixels across 60 oak-dominated forest sites spanning 2,400 km^2^ in southern Germany (Extended Data Fig. 1). The ecological dynamics of this region, including seasonally timed herbivore pressure, host-specific interactions and substantial intraspecific variation in spring phenology, are characteristic of temperate deciduous forests worldwide, as demonstrated by global syntheses of phenology–climate interactions^10,38^, classical work on temperate forest herbivore–host dynamics^17,22,39^ and cross-continental analyses of phenological variation in dominant deciduous tree species^40,41^. Data were sampled during the 5 years (2017–2021) and yielded 137,500 pixel-based observations. In 2019, a spongy moth (*Lymantria dispar*) outbreak occurred, when defoliation was experimentally suppressed in half the sites through aerial insecticide application^27^. This created a wide gradient of simultaneous herbivore pressure, ranging from high levels in outbreak sites to low levels in treated sites (Methods), against which we compared budburst timing. Using generalized additive models (GAMs) that account for undetected spatiotemporal variation (such as differences in microclimate), we addressed three key research questions:

1. Do trees delay budburst in response to prior-year herbivory (hypothesis 1) and is this delay weaker at outbreak sites (hypothesis 3)?
2. Does such a delay reduce subsequent herbivory (hypothesis 2) and is this reduction weaker at outbreak sites (hypothesis 4)?
3. Is the herbivory-driven budburst delay greatest in forests where it most efficiently reduces herbivory (hypothesis 5)?

## Results and discussion

### Leaf herbivory delays subsequent budburst

Our satellite-borne radar-based indices of canopy development revealed considerable spatiotemporal variation in budburst phenology of trees (Fig. 1b and Supplementary Video 1). This reveals a ‘phenological mosaic’ across forests—a spatially and temporally structured pattern in the timing of bud development among individual trees, conceptually analogous to the mosaic cycle theory^42^ for the development of entire trees. While spatial variation in phenology among tree individuals is well documented, our results suggest that it can organize into a cyclic spatiotemporal mosaic. These phenological mosaics provide a framework for investigating the ecological drivers of budburst timing across tree populations.

**Fig. 2:**
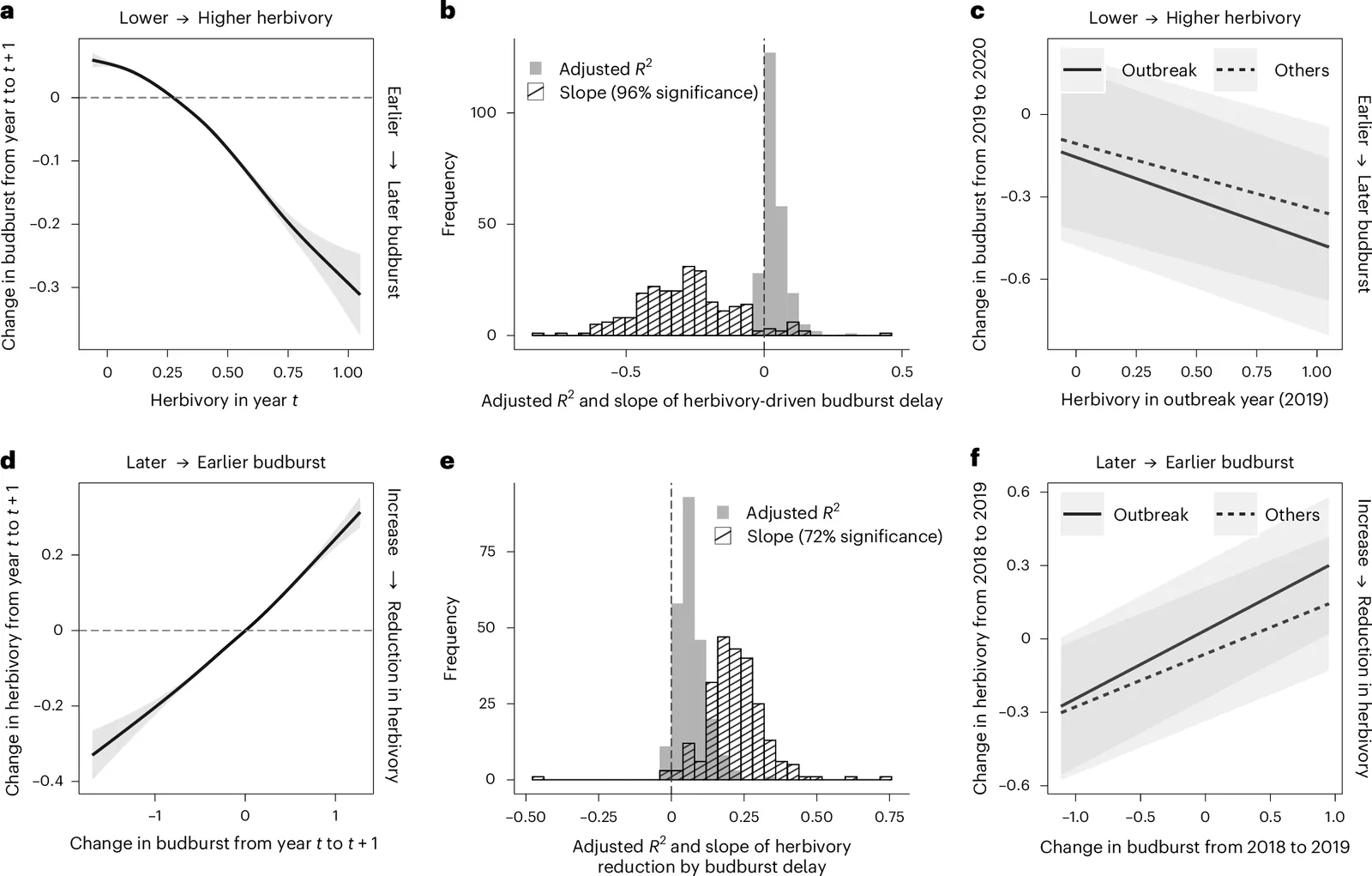
Observed relationships between budburst timing and leaf herbivory. The relationship illustrates herbivory-driven delays in budburst (top row) and herbivory reductions by delayed budburst (bottom row). Solid lines are GAM predictions of the expected mean response and shaded bands show the 95% confidence intervals around these predictions. **a**, Across all sites and years, greater leaf herbivory was associated with delayed budburst in the subsequent year. **b**, At the site level, this association varied substantially in both slope and adjusted *R*^2^. **c**, The association was somewhat stronger at sites experiencing spongy moth outbreaks. **d**, In turn, across all sites and years, delayed budburst was associated with reduced leaf herbivory. **e**, Site-level relationships again showed considerable variation in slope and adjusted *R*^2^. **f**, The effect appeared somewhat stronger at outbreak sites. Source data

Across 5 years and 60 forest sites, we tested whether leaf herbivory in year *t* predicted changes in the budburst ranks of pixels from year *t* to *t* + 1. In support of hypothesis 1, we found that higher herbivory in year *t* was related to later budburst in year *t* + 1 (*n* = 110,000, *F* = 596.5, *P* < 0.001, adjusted *R*^2^ = 0.102; Fig. 2a). This pixel-level pattern persisted even after accounting for potential effects of tree growth (Supplementary Method 1), indicating that the pattern is unlikely to result simply from herbivory depleting tree resources. To evaluate the robustness of this pattern and its generality across forest sites, we repeated tests separately for each combination of forest site and year pair (60 sites × 4 two-subsequent-year periods = 240 cases) and extracted the slope of the relationship (a negative slope indicates that higher herbivory in year *t* was associated with a later budburst in year *t* + 1). A sign test on these slopes revealed that the pattern was remarkably consistent, with 226 of the 240 slopes being negative (Fig. 2b, sign test: *P* < 0.001, success probability >0.942). Thus, our results provide evidence that, across years and forest sites spread over 2,800 km^2^, leaf herbivory in one year can result in a delayed budburst in the subsequent year. Despite the consistency in direction, the magnitude of this pattern varied widely among forest sites (Fig. 2b), with site-level explained variance (adjusted *R*^2^) ranging from −0.020 to 0.322.

The average magnitude of budburst delay was remarkable: on average, buds on trees exposed to above-median prior-year herbivory opened 3 days later than those with below-median prior-year herbivory (Supplementary Methods 2). Long-term studies have shown that elevated spring temperatures advance budburst in temperate trees by 2.5 days per decade^8–12^. Hence, our data suggest that a single year of elevated herbivory can counteract, and even exceed, the phenological effect of a decade of climate warming. This antagonism may help explain a persistent puzzle: budburst advancement often lags behind the rate of warming^12–14^. It should be noted that most phenological models still project continued advancement of budburst under climate change^5,7,8,12,43^, while rarely, if ever, accounting for biotic interactions such as herbivory—a major omission, given our findings and the fact that insect populations and herbivory seem to be consistently changing^44–46^.

We expected a weakened phenological response in outbreak sites, assuming that outbreak herbivory would provide a less reliable signal about risk of future herbivory (hypothesis 3)^21,32^. To test this, we analysed the change in budburst timing from 2019 (the outbreak year) to 2020 as a function of 2019 herbivory, an outbreak factor (affected versus unaffected sites) and their interaction. We focused on the interaction to assess whether the relationship between herbivory and delayed budburst differed between site types. Contrary to our expectation (hypothesis 3), the response was stronger at outbreak sites (*n* = 27,500, *t* = −3.588, *P* < 0.001; Fig. 2c). Trees experiencing outbreak herbivory even showed somewhat greater delays in budburst than trees from non-outbreak sites, suggesting that trees may not detect or respond to landscape-level signals. Instead, their response appears to be primarily driven by their own herbivory.

### Budburst delay reduces leaf herbivory

Using the same 5-year, 60-site dataset, we next tested whether changes in relative budburst timing from year *t* to *t* + 1 of satellite pixels predicted changes in leaf herbivory over the same interval. In support of hypothesis 2, herbivory decreased as budburst timing was delayed (*n* = 110,000, *F* = 1593.2, *P* < 0.001, adjusted *R*^2^ = 0.149; Fig. 2d). This relationship remained robust after accounting for potential herbivore control by natural antagonists such as parasitoids and viruses and abiotic factors as inferred from indicator values of ground vegetation (Supplementary Methods 3). Again, the pattern proved to be consistent, occurring in 236 out of 240 site–year combinations (Fig. 2e; sign test *P* < 0.001, success probability >0.983). Thus, our results provide robust and large-scale support for the hypothesis that delayed budburst consistently reduces leaf herbivory by insects. Despite the consistency in direction, the magnitude of this pattern also varied substantially among forest sites (Fig. 2e), with site-level explained variance (adjusted *R*^2^) ranging from −0.015 to 0.321.

**Extended Data Fig. 2:**
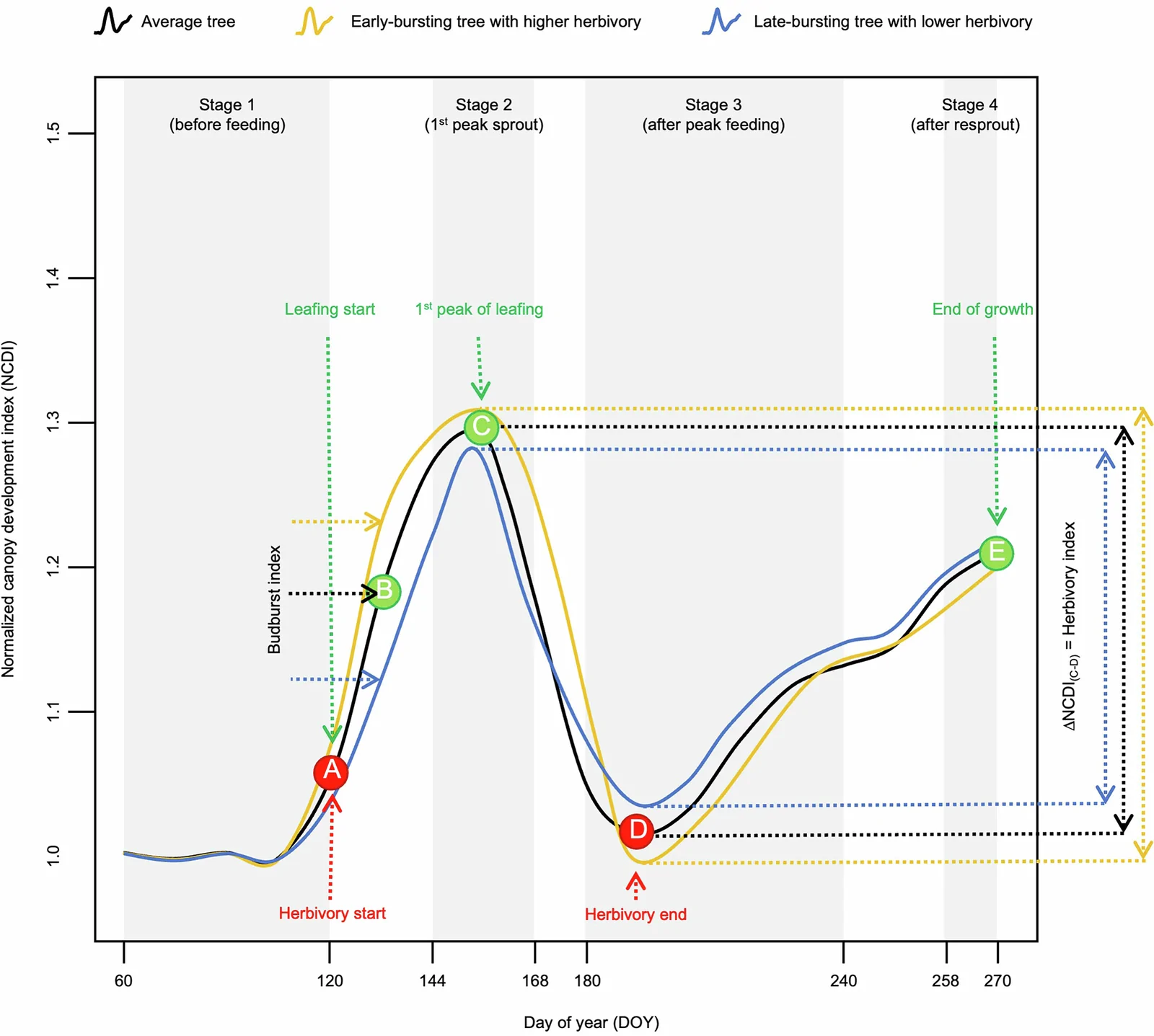
Exemplary illustration of the use of the normalized canopy development index (NCDI) as a measure of budburst phenology and herbivory impact. The diagram is adapted from Bae et al.^37^, and represents the temporal trajectory of NCDI values at single-day level. Note that the DOY values in the x-axis are representative for the years 2017 and 2019, and the growing season was relatively early in 2018 and 2020 and relatively late in 2021. To account for such interannual variations, analyses were conducted with relative measures of budburst timing (see Methods for details).

The average magnitude of herbivory reduction was remarkable. On average, pixels that delayed budburst profited from a 55% reduction in herbivory compared with the previous year (Supplementary Methods 2). Given the broad seasonal window of herbivore activity in the region (~60 days; Extended Data Fig. 2), such a substantial reduction resulting from only a 3-day delay in budburst may seem surprising at first. However, a short delay can substantially increase herbivore mortality among the first larval instars, which are particularly vulnerable. This is supported by experimental studies showing that herbivores hatching just 3 days earlier than budburst exhibited mortality rates up to 90% (refs. ^47,48^). By comparison, achieving a 50% reduction in herbivory via the chemical defence of trees would require an approximately six-fold increase in tannin concentration^17,49,50^. This highlights that herbivory-induced budburst delay may represent an effective defence strategy that equates or even exceeds the benefits of induced chemical defence and of constitutive defences that are produced even in the absence of attack^36,50,51^. It may even be more potent than the effect of natural antagonists of the spongy moth, given their comparatively low impact (Supplementary Methods 3).

Our findings support the hypothesis that delayed budburst after herbivory, a strategy recently described as ‘escaping enemies in time’^16^, disrupts the phenological synchrony between host trees and their herbivores^11,15,21,31^. By postponing leaf emergence, trees reduce the access of early-season herbivores to young, nutrient-rich foliage^17^. This phenological mismatch can suppress herbivore performance by delaying development, reducing feeding efficiency and increasing mortality^11,15,21,31^. Moreover, delayed budburst is often associated with accelerated leaf maturation^17,22^ so that leaves quickly enhance structural defences and chemical resistance, which further diminishes herbivore success. However, the effects of budburst delay may vary among herbivore communities. In temperate deciduous forests, herbivore communities are typically dominated by spring-feeding Lepidoptera whose first instars have narrow temporal windows of high vulnerability^11,17,21,39,52^. By contrast, another fraction of the herbivore community has broader temporal niches, is less constrained by short delays in budburst and tends to contribute proportionally more to later-season herbivory^21,22^. Therefore, the among-site variation of the magnitude to which budburst delay reduces herbivory may reflect the composition and flexibility of the local herbivore community^16,21^.

We then assessed whether the relationship between budburst delay and herbivory reduction weakened in outbreak sites (hypothesis 4). We therefore tested for a negative interaction term between outbreak (affected versus unaffected sites) and budburst delay affecting the reduction of herbivory from 2018 to 2019 (the outbreak year). Contrary to our expectation (hypothesis 4), we found a positive interaction term, meaning the relationship was stronger in outbreak sites (*n* = 27,500, *t* = 5.768, *P* < 0.001; Fig. 2f) and this relationship remained robust after accounting for potential herbivore control by natural antagonists (Supplementary Methods 3). The observation that trees from outbreak sites with delayed budburst experienced reduced herbivory challenges the common assumption that severe herbivore outbreaks overwhelm tree defences^26–28,31^—a scenario in which even trees that delay budburst would be expected to suffer severe defoliation under extreme herbivore densities^21,31,32^. A likely explanation for the persistence of this pattern under outbreak conditions is that spongy moth populations are particular in that they often decline rapidly as a result of pathogen epizootics after reaching their peak^27,28^, narrowing the window of intense feeding pressure. Trees that delay budburst may therefore avoid the most damaging window of this type of herbivore activity. This mechanism would also explain why some forest sites with very high egg-mass densities, predicting near-complete canopy loss^27,37^, actually experience low levels of defoliation in that year. Together, these findings suggest that phenological asynchrony remains an ecologically effective defence strategy, even during large-scale insect outbreaks.

### Adaptive coupling of delay and defence

**Fig. 3:**
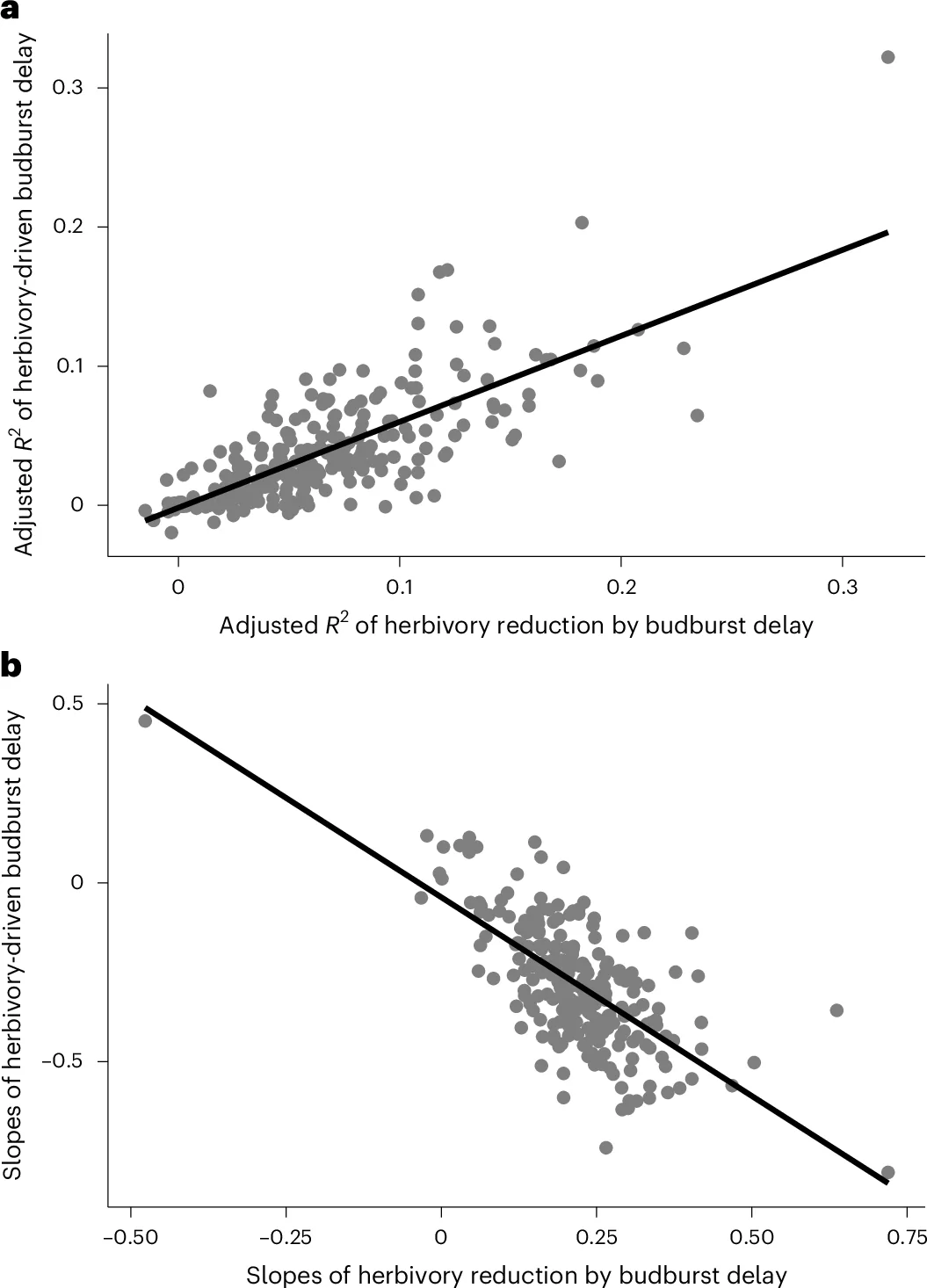
Coupling between the strength of herbivory-driven budburst delay and the strength of herbivory reduction by budburst delay, analysed across forest sites. **a**,**b**, The strengths of the relationships are interpreted from the site-level adjusted *R*^2^ values (**a**) and slope values (**b**). Note that relationships are expected to be positive in **a** and negative in **b**. The strength of herbivory-driven budburst delay increases with the strength of that delay in reducing subsequent herbivory, suggesting that trees delay budburst more strongly in forest sites where this strategy is more effective. This pattern helps to explain why herbivory induces much greater delays in some forests than in others (Fig. 2b). Source data

Herbivory-driven budburst delay could be proximately explained by physiological constraints such as resource depletion^4,36,53^ and hormonal regulation^54,55^, but these proximate explanations do not preclude the possibility that a physiologically induced delay is also an adaptive plastic response ultimately shaped by natural selection retaining more plastic genotypes within populations. Direct experimental tests of adaptation would require long-term, whole-tree manipulations of herbivory until fitness differences translate into changes of allele frequencies among descendants. This is infeasible. Instead, we asked whether the strongest herbivory-driven budburst delays occur in forests where such delays yield the greatest reduction in herbivory and, thus, the greatest potential fitness advantage. To test this, we used the explained variance (adjusted *R*^2^) of the previously described site–year specific analyses, regressing the strength of herbivory-driven budburst delay against the strength of herbivory reduction by budburst delay. Across 240 site–year cases, these two metrics were positively correlated (d.f. = 235, *t* = 16.769, *P* < 0.001; Fig. 3a), supporting hypothesis 5. When we repeated the analysis using slopes, an alternative measure of relationship strength, we again found a strong correlation (d.f. = 235, *t* = −14.219, *P* < 0.001; Fig. 3b). Note that, as expected, the slopes are opposite in sign: trees exhibiting the greatest budburst delay in response to prior herbivory (negative slopes) occurred in forests where delayed budburst most effectively reduced subsequent herbivory (positive slopes). These site-level patterns of adjusted *R*^2^ remained robust (in every case *P* < 0.001, *t* = 4.513–11.951) even after controlling for potential effects of tree growth, herbivore regulation by parasitoids or viruses, or abiotic conditions (as inferred from indicator values of herbaceous vegetation; Supplementary Methods 3). Furthermore, the analyses had a higher adjusted *R*^2^ (0.548) when none of these confounding factors was included than when one of the possible confounders was accounted for (0.347 to 0.480). Therefore, the delay of budburst with herbivory across dozens of tree populations suggests that budburst delay is not only a physiological response by individual trees to prior-year herbivory, or of natural enemies, but an adaptation selected in those tree populations where such a delay increases fitness most^35,36,43,56,57^.

Overall, in forest sites where herbivore emergence is tightly timed to coincide with early budburst, even a modest delay can substantially reduce damage and we may speculate that this increases tree performance, especially in young trees. The resulting population of adult trees would show a high proportion of ‘budburst-delayers’ and a strong relationship between prior herbivory and budburst timing. Trees appear to choose to adjust their phenology on the basis of recent herbivory, hence balancing^34–36^ the benefits of herbivore escape against the possible costs associated with a shortened growing season^43,58^. Because such costs may reduce competitive dominance, trees often avoid delaying budburst to mitigate these effects. Taken together, these findings are consistent with a scenario of budburst delay as a case of adaptive plasticity that evolved under natural selection due to herbivore pressure.

### Possible implications

The dynamic nature of budburst phenology revealed here may help to stabilize tree populations by mitigating recurrent herbivore damage and promoting demographic resilience, while also shaping herbivore community dynamics in forest ecosystems^59,60^. In forests where relative budburst timing is fixed, early-bursting trees would consistently face higher herbivory, reducing their fitness, depleting early-bursting genotypes and eroding phenological diversity within populations. Phenological mosaics (as seen in Fig. 1b and Supplementary Video 1) disrupt this asymmetry by introducing a delayed negative feedback: trees that are heavily defoliated in one year tend to postpone budburst the next year, thereby escaping peak herbivore pressure. This redistribution of herbivory across individuals and years could buffer extreme outcomes, prevent localized perennial overexploitation and maintain within-population variation in phenology between currently delayed and non-delayed plastic trees and between plastic and non-plastic trees. Although this strategy primarily benefits trees, it may also sustain herbivore diversity by making host phenologies less predictable^20,61^. Delayed budburst can disadvantage herbivores that are tightly synchronized with predictable host timing^11,17,21,39,52^, while opening temporal niches for generalist species that are less reliant on synchronous egg hatching^21,22^. Such spatiotemporal heterogeneity in resource availability arising from phenological mosaics of trees could weaken competitive exclusion among herbivores and promote coexistence^53,62,63^. Importantly, because these budburst delays are transient and contingent on prior-year herbivory, they offer little opportunity for herbivores to adaptively shift their own phenology. Moreover, herbivore developmental timing is strongly constrained by climatic cues, which, under ongoing warming, generally favour earlier rather than later emergence. As a result, the intermittent and reversible budburst delays documented here are unlikely to drive long-term adaptive shifts in herbivore phenology.

Beyond these community-level consequences, our results also point to a mechanism for delayed density dependence in herbivore populations. For decades, population cycles in forest Lepidoptera have been attributed to delayed density-dependent effects mediated by natural antagonists^62,63^ (for example, parasitoids and viruses) or, more hypothetically, by induced plant chemical defences^64,65^. However, direct evidence for the latter has been limited. Our analyses show that budburst-mediated reductions of herbivory are both consistent and, in strength, exceed those attributable to natural antagonists (Supplementary Methods 3). This suggests that delayed responses in tree phenology following herbivory can act as a population-level negative feedback mechanism. By reducing herbivore success with a one-year lag, budburst delay may generate the ‘second-order’ dynamics^62–65^ long recognized as being central to herbivore population oscillations. These results demonstrate a phenology-driven pathway to delayed density dependence, offering a realistic alternative or complement to the classical mechanisms based on natural antagonists and chemical defences.

## Conclusions and outlook

Our findings demonstrate that herbivory-induced delays in budburst constitute a widespread and effective defence strategy, even during extreme outbreaks of herbivores. Moreover, our results suggest that herbivory-induced phenological mosaics enhance forest resilience by dynamically redistributing herbivore pressure across tree individuals and years. Although our study is geographically focused on oak-dominated forests in southern Germany, the ecological dynamics underpinning this mechanism, such as seasonally timed herbivore pressure, host-specific interactions and intraspecific phenological variation are common across temperate deciduous forests globally. As such, we anticipate that similar time-based escape strategies may operate in other systems with tightly coupled phenological relationships. Moreover, our study opens several directions for future research. Our consistently observed patterns across forest sites, years and outbreak conditions point to a functional link between delayed budburst and reduced herbivore damage. To isolate the role of budburst timing from other traits, future studies should adopt manipulative experimentation, such as modulating the phenology of trees or excluding herbivores. However, such experiments will inevitably have to be carried out at a smaller scale and would need to ensure that herbivore populations are synchronized with their host tree individuals before experimentation. Finally, our study highlights that trees are experiencing opposing selection pressures from climate warming and herbivory, which may place them at risk of being caught in an evolutionary trap. Hence, we suggest that it is only possible to fully understand tree response to abiotic changes—such as why budburst phenology often lags behind climate warming—by also considering biotic interactions.

## Methods

### Study site, herbivore outbreak and manipulation of herbivore load

**Extended Data Fig. 3:**
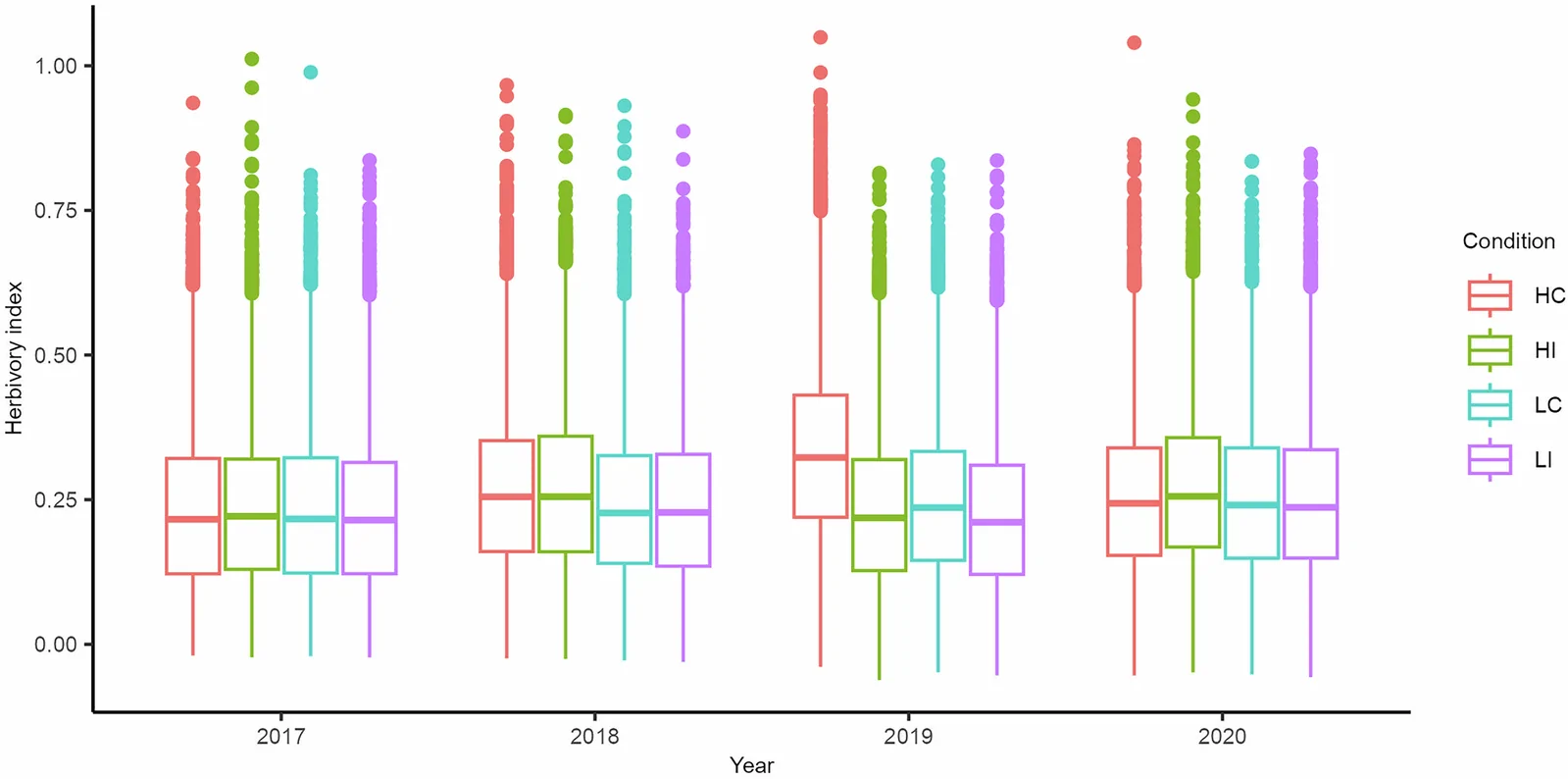
Distribution of herbivory index across years and treatment conditions. The four treatment combinations (see Methods for details) comprised high defoliation risk unsprayed control sites (HC), high defoliation risk insecticide-treated sites (HI), low defoliation risk unsprayed control sites (LC), and low defoliation risk insecticide-treated sites (LI). Herbivory was quantified for each pixel from satellite data. During the outbreak year 2019, herbivory was only elevated in HC sites (that is, sites with high egg mass densities but not treated with insecticides). Despite the generally higher population levels in HC sites in 2019 (the outbreak year), there was substantial variation in herbivory among trees.

The study was conducted across 60 forest sites in Franconia, Bavaria, Germany (200–500 m a.s.l., centred at 49° 37′ N, 10° 24′ E, spanning over a landscape of 2,400 km^2^ as shown in Extended Data Fig. 1). Each forest site covered an average area of 4.6 ha (s.d*.* ±2.1) and experienced a temperate climate, with mean annual temperatures ranging from 7.5 °C to 9.0 °C and annual precipitation between 600 mm and 1,000 mm. The forests are dominated by *Quercus robur* and *Quercus petraea*, which have periodically undergone *L. dispar* outbreaks since the early 1990s^28^. During our study period (2017–2021), 2019 was identified as an outbreak year, on the basis of the defoliation risk index (DRI), calculated from mean *L. dispar* egg-mass densities at forest sites compared with critical egg-mass density thresholds (details in refs. ^27,37^). Although some forest localities experienced high DRI levels due to outbreak conditions as predicted for 2019, others remained at background DRI levels; giving us two defoliation risk levels—high (H) and low (L). Within each defoliation risk level, half the forest localities were randomly selected for herbivore-load reduction by spraying insecticide (I): tebufenozide aerially sprayed between 3 and 23 May 2019, while the other half remained unsprayed as a control (C). This 2 × 2 factorial design resulted in four treatment combinations: HC, HI, LC and LI. HC sites experienced elevated herbivory than all other sites (Extended Data Fig. 3); therefore we classified HC sites as outbreak-affected and remaining sites as non-affected.

### Remote sensing of budburst phenology and herbivory impact

To monitor budburst phenology and herbivory, we followed the methods of ref. ^37^, in applying Sentinel-1 satellite data (10 × 10 m^2^ spatial resolution per pixel which represents the size of average trees crows) to quantify the dynamics of insect herbivory in high temporal resolution. Data of Sentinel-1 C-band synthetic aperture radar were acquired from ESA Scientific Hub for March–September of 2017–2021, using all available level-1 ground-range-detected high-resolution products. These 10-m pixel dual-polarization (VV and VH) data were preprocessed using ESA SNAP (Sentinel Application Platform) v.7.0 with the Sentinel-1 Toolbox, including precise orbit correction, thermal noise removal, radiometric calibration to *β*^0^, radiometric terrain flattening and range-Doppler terrain correction to convert *β*^0^ to *γ*^0^. The log-transformed *γ*^0^ time series were smoothed using a Gaussian filter implemented in the smoother package in R v.4.2.1, with raster data processing and interpolation handled using the raster and zoo packages, to produce single-day smoothed series per pixel and polarization. These per-pixel single-day *γ*^0^ values for VV and VH were used to characterize forest canopy status. The canopy development index (CDI = *γ*^0^_VV_ − *γ*^0^_VH_) was then computed because differences between VV and VH temporal profiles effectively identify deciduous forest phenology^66^. To reduce confounding influences such as species composition or soil conditions, the normalized CDI (NCDI) was obtained by dividing the CDI time series of each pixel by its minimum leaf-off CDI value^37^. The temporal trajectory of the NCDI represents the amount of foliage present in the canopy over time, allowing us to identify key phenological transitions (Extended Data Fig. 2). Five major phenological transitions were conceptualized from NCDI time series spanning the tree-growing season. Step A marks the onset of budburst and canopy herbivory, step B the midpoint of budburst, step C the first peak of leafing (greening), step D the end of canopy herbivory and step E the end of the growing season (Extended Data Fig. 2; ref. ^37^).

By analysing the temporal trajectory of NCDI, we derived two key metrics related to budburst phenology and herbivore impact: the budburst index (equation (1)) and the herbivory index (equation (2)). The budburst index represents the NCDI value at the midpoint of leaf flushing, marked by step B. Because the timing of the phenological window varied among years (Extended Data Fig. 2), we conducted analyses based on relative budburst measures. Specifically, within each year, the minimum budburst index was rescaled to zero by subtracting the lowest NCDI value at step B from all individual pixel values in that year. This procedure yields a relative budburst ranking (for simplicity, we retain the term budburst index), in which higher values indicate earlier budburst relative to the latest tree within a given year. This normalization enables direct comparisons across years. We calculated the herbivory index that is the extent of defoliation caused by herbivory as the difference in NCDI values between steps C and D (Extended Data Fig. 2). Using these indices, we further assessed interannual changes in budburst phenology (equation (3)) and changes in herbivory impact (equation (4)). Changes in budburst phenology were determined by subtracting the budburst index of the previous year from the value for each pixel of the current year, with positive values indicating an advancement in budburst timing. Similarly, changes in herbivory impact were obtained by subtracting the herbivory index of the current year from the previous years, where positive values signify an increase in defoliation compared with the prior year (*y* below).

1 $${\rm{Budburst}}\,{\rm{index}}={{\rm{NCDI}}}_{{\rm{step}}\,{\rm{B}}}$$

2 $${\rm{Herbivory}}\,{\rm{index}}={{\rm{NCDI}}}_{{\rm{step}}\,{\rm{C}}}-{{\rm{NCDI}}}_{{\rm{step}}\,{\rm{D}}}$$

3 $${{\rm{Changes}}\,{\rm{in}}\,{\rm{budburst}}={\rm{Budburst}}\,{\rm{index}}}_{y=n+1}-{{\rm{Budburst}}\,{\rm{index}}}_{y=n}$$

4 $${{\rm{Changes}}\,{\rm{in}}\,{\rm{herbivory}}={\rm{Herbivory}}\,{\rm{index}}}_{y=n}-{{\rm{Herbivory}}\,{\rm{index}}}_{y=n+1}$$

Satellite-derived measurements of budburst phenology and herbivory offer broad spatial and temporal coverage but inevitably involve estimation and added noise compared with direct field observations. Others^37^ developed both 12-day composite and single-day smoothed NCDI time series and the single-day measures derived from several stages of the temporal trajectory were validated against optical (Sentinel-2) and terrestrial laser scanning data (Fig. 2 of ref. ^37^), supporting confidence in our single-day budburst index. In that study^37^, Sentinel-2 data were aggregated at the forest site scale (4.6 ha), while terrestrial laser scanning data were aggregated over 0.5 ha at site centres. Moreover, our herbivory index was validated at the level of forest sites (4.6 ha), using intensive caterpillar sampling from canopy fogging (Fig. 3 and Supplementary Fig. 7 of ref. ^37^). In the present study, we added a finer, pixel-level evaluation, again confirming the reliability of the herbivory index. For this purpose, we studied pixels from the outbreak year (2019) that had high herbivore densities as inferred from high numbers of egg masses measured in the field. We tested whether, independent of site identity, the pixels that were treated by aerial insecticide application had lower herbivory index values than pixels that had not been treated. We confirmed that this was the case (Extended Data Fig. 3; linear mixed-effects model *z* = 18.422, *P* < 0.001). Furthermore, in the preceding years when no insecticide was applied, the same two groups of pixels did not differ (*z* = −0.336 and −0.486, both *P* ≈ 1). Note that Sentinel-1 is cloud independent, little prone to saturation contrary to both Sentinel-2 and Planetscope and corrected for effects of canopy structure, background reflectance and atmospheric conditions^37,67^. Nonetheless, it may still contain errors that are not systematic biases capable of generating patterns, but rather random noise that can obscure true patterns. Despite this noise, the emergence of patterns consistent with ecological hypotheses strengthens our confidence in their relevance.

### Statistical analyses

To test whether herbivore attacks lead to budburst delay (question 1), we fitted GAMs optimized for large datasets using the bam function in the mgcv package^68^ in R v.4.5.1^69^. Across 60 sites and 5 years, we modelled year-to-year ‘changes in budburst’ (defined as changes in the budburst rank of an individual pixel relative to other pixels) as a function of prior-year ‘herbivory index’. Spatiotemporal variation was accounted for by including longitude and latitude as penalized regression spline smoothers and study year and experimental block (Extended Data Fig. 1) as random-effect smoothers. To test robustness and consistency across sites, we also fitted site–year-specific models and conducted a sign test on resulting slopes. To assess whether the relationship persisted during outbreak-level herbivory, we extended the model to focus on 2019 (outbreak year), testing whether herbivory indices of 2019 predicted changes in budburst between 2019 and 2020 and included an outbreak factor (outbreak-affected versus non-affected sites) and its interaction with herbivory indices of 2019.

Similarly, to test whether budburst delay reduces herbivory (question 2), we again used GAMs. Here ‘changes in herbivory’ were modelled as a function of changes in budburst. Spatiotemporal variation was controlled as above and robustness and consistency were assessed using site–year-specific models and a sign test. To evaluate outbreak effects, we focused on 2018–2019 transitions, including an outbreak factor (high or low DRI) and its interaction with ‘changes in budburst’.

Finally, to test whether budburst delay was stronger in forest sites where it more effectively reduced herbivory (question 3), we regressed the explained variance (adjusted *R*^2^) of site–year-specific models for question 1 against those for question 2, with study year as a covariate and forest site as a random factor.

### Reporting summary

Further information on research design is available in the Nature Portfolio Reporting Summary linked to this article.

## Supplementary information

Supplementary Information Supplementary Video caption, Methods 1–3 and References.

Reporting Summary

Peer Review File

Supplementary Video 1 Interannual shift in budburst phenology. This video illustrates year-to-year shifts in budburst timing among trees in the four treatment plots (HC, HI, LC and LI) within block A. Satellite-detected pixels are colour-coded on the basis of relative budburst timing: white (late) → green (early). The study year is indicated in the top-left corner of each frame.

## Source data

Source Data Fig. 2 Statistical source data.

Source Data Fig. 3 Statistical source data.

## Data Availability

The data are available via Zenodo at https://doi.org/10.5281/zenodo.17285429 (ref. ^70^). Source data are provided with this paper.

## Code availability

The code is available via Zenodo at https://doi.org/10.5281/zenodo.17285429 (ref. ^70^).

## References

[1] Basler, D. & Körner, C. Photoperiod sensitivity of bud burst in 14 temperate forest tree species. *Agric. For. Meteorol.* **165**, 73–81 (2012).

[2] Basler, D. & Körner, C. Photoperiod and temperature responses of bud swelling and bud burst in four temperate forest tree species. *Tree Physiol.* **34**, 377–388 (2014). 10.1093/treephys/tpu021 24713858

[3] Jochner, S. et al. Nutrient status: a missing factor in phenological and pollen research?. *J. Exp. Bot.* **64**, 2081–2092 (2013). 10.1093/jxb/ert061 PMC3638828 23630329

[4] Marchand, L. J. et al. Inter-individual variability in spring phenology of temperate deciduous trees depends on species, tree size and previous year autumn phenology. *Agric. For. Meteorol.* **290**, 108031 (2020). 10.1016/j.agrformet.2020.108031 PMC7304479 32817727

[5] Chuine, I., Cour, P. & Rousseau, D. D. Selecting models to predict the timing of flowering of temperate trees: implications for tree phenology modelling. *Plant Cell Environ.* **22**, 1–13 (1999).

[6] Piao, S. et al. Plant phenology and global climate change: current progresses and challenges. *Glob. Change Biol.* **25**, 1922–1940 (2019). 10.1111/gcb.14619 30884039

[7] Pau, S. et al. Predicting phenology by integrating ecology, evolution and climate science. *Glob. Change Biol.* **17**, 3633–3643 (2011).

[8] Peñuelas, J. & Filella, I. Responses to a warming world. *Science* **294**, 793–795 (2001). 10.1126/science.1066860 11679652

[9] Parmesan, C. & Yohe, G. A globally coherent fingerprint of climate change impacts across natural systems. *Nature* **421**, 37–42 (2003). 10.1038/nature01286 12511946

[10] Menzel, A. et al. European phenological response to climate change matches the warming pattern. *Glob. Change Biol.* **12**, 1969–1976 (2006).

[11] Visser, M. E. & Holleman, L. J. M. Warmer springs disrupt the synchrony of oak and winter moth phenology. *Proc. R. Soc. B* **268**, 289–294 (2001). 10.1098/rspb.2000.1363 PMC1088605 11217900

[12] Jeong, S.-J., Ho, C.-H., Gim, H.-J. & Brown, M. E. Phenology shifts at start vs. end of growing season in temperate vegetation over the Northern Hemisphere for the period 1982–2008. *Glob. Change Biol.* **17**, 2385–2399 (2011).

[13] Fu, Y. H. et al. Declining global warming effects on the phenology of spring leaf unfolding. *Nature* **526**, 104–107 (2015). 10.1038/nature15402 26416746

[14] Wenden, B., Mariadassou, M., Chmielewski, F.-M. & Vitasse, Y. Shifts in the temperature-sensitive periods for spring phenology in European beech and pedunculate oak clones across latitudes and over recent decades. *Glob. Change Biol.* **26**, 1808–1819 (2020). 10.1111/gcb.14918 31724292

[15] Ren, P. et al. Warming counteracts defoliation-induced mismatch by increasing herbivore–plant phenological synchrony. *Glob. Change Biol.* **26**, 2072–2080 (2020). 10.1111/gcb.14991 31925858

[16] Mallick, S. et al. Unapparent trees: escaping enemies in time by being discreet, unpredictable and inaccessible. *Oikos* **2025**, e11218 (2025).

[17] Feeny, P. Seasonal changes in oak leaf tannins and nutrients as a cause of spring feeding by winter moth caterpillars. *Ecology* **51**, 565–581 (1970).

[18] Quiring, D. T. & McKinnon, M. L. Why does early-season herbivory affect subsequent budburst?. *Ecology* **80**, 1724–1735 (1999).

[19] Lowman, M. D. & Box, J. D. Variation in leaf toughness and phenolic content among five species of Australian rain forest trees. *Aust. J. Ecol.* **8**, 17–25 (1983).

[20] Carroll, A. L. & Quiring, D. T. Herbivory modifies conifer phenology: induced amelioration by a specialist folivore. *Oecologia* **136**, 88–95 (2003). 10.1007/s00442-003-1240-5 12720084

[21] Van Asch, M. & Visser, M. E. Phenology of forest caterpillars and their host trees: the importance of synchrony. *Annu. Rev. Entomol.* **52**, 37–55 (2007). 10.1146/annurev.ento.52.110405.091418 16842033

[22] Hunter, M. D. A variable insect–plant interaction: the relationship between tree budburst phenology and population levels of insect herbivores among trees. *Ecol. Entomol.* **17**, 91–95 (1992).

[23] Shutt, J. D., Burgess, M. D. & Phillimore, A. B. A spatial perspective on the phenological distribution of the spring woodland caterpillar peak. *Am. Nat.* **194**, E109–E121 (2019). 10.1086/705241 31613670

[24] Visser, M. E., Holleman, L. J. M. & Gienapp, P. Shifts in caterpillar biomass phenology due to climate change and its impact on the breeding biology of an insectivorous bird. *Oecologia* **147**, 164–172 (2006). 10.1007/s00442-005-0299-6 16328547

[25] Mlynarek, J. J. et al. Enemy escape: a general phenomenon in a fragmented literature?. *FACETS* **2**, 1015–1044 (2017).

[26] Seidl, R. et al. Forest disturbances under climate change. *Nat. Clim. Change* **7**, 395–402 (2017). 10.1038/nclimate3303 PMC5572641 28861124

[27] Leroy, B. M. L. et al. Relative impacts of gypsy moth outbreaks and insecticide treatments on forest resources and ecosystems: an experimental approach. *Ecol. Solut. Evid.* **2**, e12045 (2021).

[28] Lemme, H., Lobinger, G. & Müller-Kroehling, S. SchwammspinnerMassenvermehrung in Franken. *LWF Aktuell* **121**, 37–43 (2019).

[29] Moreno, J. E., Tao, Y., Chory, J. & Ballaré, C. L. Ecological modulation of plant defense via phytochrome control of jasmonate sensitivity. *Proc. Natl Acad. Sci. USA* **106**, 4935–4940 (2009). 10.1073/pnas.0900701106 PMC2660767 19251652

[30] Rasheed, M. U., Brosset, A. & Blande, J. D. Tree communication: the effects of ‘wired’ and ‘wireless’ channels on interactions with herbivores. *Curr. For. Rep.* **9**, 33–47 (2023).

[31] Pureswaran, D. S. et al. Climate-induced changes in host tree–insect phenology may drive ecological state-shift in boreal forests. *Ecology* **96**, 1480–1491 (2015).

[32] Jepsen, J. U., Hagen, S. B., Ims, R. A. & Yoccoz, N. G. Climate change and outbreaks of the geometrids *Operophtera brumata* and *Epirrita autumnata* in subarctic birch forest: evidence of a recent outbreak range expansion. *J. Anim. Ecol.* **77**, 257–264 (2008). 10.1111/j.1365-2656.2007.01339.x 18070041

[33] White, J. A. & Whitham, T. G. Associational susceptibility of Cottonwood to a Box Elder herbivore. *Ecology* **81**, 1795–1803 (2000).

[34] Bennie, J., Kubin, E., Wiltshire, A., Huntley, B. & Baxter, R. Predicting spatial and temporal patterns of bud-burst and spring frost risk in north-west Europe: the implications of local adaptation to climate. *Glob. Change Biol.* **16**, 1503–1514 (2010).

[35] Gauzere, J. et al. Where is the optimum? Predicting the variation of selection along climatic gradients and the adaptive value of plasticity. A case study on tree phenology. *Evol. Lett.* **4**, 109–123 (2020). 10.1002/evl3.160 PMC7156102 32313687

[36] Herms, D. A. & Mattson, W. J. The dilemma of plants: to grow or defend. *Quart. Rev. Biol.* **67**, 283–335 (1992).

[37] Bae, S. et al. Tracking the temporal dynamics of insect defoliation by high-resolution radar satellite data. *Methods Ecol. Evol.* **13**, 121–132 (2022).

[38] Cleland, E. E., Chuine, I., Menzel, A., Mooney, H. A. & Schwartz, M. D. Shifting plant phenology in response to global change. *Trends Ecol. Evol.* **22**, 357–365 (2007). 10.1016/j.tree.2007.04.003 17478009

[39] Forkner, R. E., Marquis, R. J., Lill, J. T. & Corff, J. L. Timing is everything? Phenological synchrony and population variability in leaf-chewing herbivores of *Quercus*. *Ecol. Entomol.* **33**, 276–285 (2008).

[40] Vitasse, Y., Porté, A. J., Kremer, A., Michalet, R. & Delzon, S. Responses of canopy duration to temperature changes in four temperate tree species: relative contributions of spring and autumn leaf phenology. *Oecologia* **161**, 187–198 (2009). 10.1007/s00442-009-1363-4 19449036

[41] Kramer, K. Phenotypic plasticity of the phenology of seven European tree species in relation to climatic warming. *Plant Cell Environ.* **18**, 93–104 (1995).

[42] Remmert, H. in *The Mosaic-Cycle Concept of Ecosystems* (ed. Remmert, H.) 1–21 (Springer, 1991).

[43] Lin, J. et al. A model of the within-population variability of budburst in forest trees. *Geosci. Model Dev.* **17**, 865–879 (2024).

[44] van Klink, R. et al. Disproportionate declines of formerly abundant species underlie insect loss. *Nature* **628**, 359–364 (2024). 10.1038/s41586-023-06861-4 PMC11006610 38123681

[45] van Klink, R. et al. Meta-analysis reveals declines in terrestrial but increases in freshwater insect abundances. *Science* **368**, 417–420 (2020). 10.1126/science.aax9931 32327596

[46] Kozlov, M. V. & Zvereva, E. L. Changes in the background losses of woody plant foliage to insects during the past 60 years: are the predictions fulfilled?. *Biol. Lett.* **11**, 20150480 (2015). 10.1098/rsbl.2015.0480 PMC4528457 26179805

[47] van Dis, N. E. et al. Phenological mismatch affects individual fitness and population growth in the winter moth. *Proc. Biol. Sci.* **290**, 20230414 (2023). 10.1098/rspb.2023.0414 PMC10445013 37608720

[48] Tikkanen, O.-P. & Julkunen-Tiitto, R. Phenological variation as protection against defoliating insects: the case of *Quercus robur* and *Operophtera brumata*. *Oecologia* **136**, 244–251 (2003). 10.1007/s00442-003-1267-7 12728310

[49] Forkner, R. E., Marquis, R. J. & Lill, J. T. Feeny revisited: condensed tannins as anti-herbivore defences in leaf-chewing herbivore communities of *Quercus*. *Ecol. Entomol.* **29**, 174–187 (2004).

[50] Gershenzon, J. in *Insect–Plant Interactions* (ed. Bernays, E. A.) Ch. 5 (CRC, 1994).

[51] Cipollini, D., Walters, D. & Voelckel, C. in *Annual Plant Reviews* (eds Voelckel, C. & Jander, G.) 263–307 (Wiley, 2014).

[52] Wagenhoff, E., Blum, R., Engel, K., Veit, H. & Delb, H. Temporal synchrony of *Thaumetopoea processionea* egg hatch and *Quercus robur* budburst. *J. Pest Sci.* **86**, 193–202 (2013).

[53] Chapin III, F. S., Schulze, E. & Mooney, H. A. The ecology and economics of storage in plants. *Annu. Rev. Ecol. Evol. Syst.* **21**, 423–447 (1990).

[54] Deng, J., Deng, X., Yao, H., Ji, S. & Dong, L. Gibberellins play an essential role in the bud growth of *Petunia hybrida*. *Curr. Issues Mol. Biol.* **46**, 9906–9915 (2024). 10.3390/cimb46090590 PMC11430761 39329942

[55] Dinh, S. T., Baldwin, I. T. & Galis, I. The HERBIVORE ELICITOR-REGULATED1 gene enhances abscisic acid levels and defenses against herbivores in *Nicotiana attenuata* plants. *Plant Physiol.* **162**, 2106–2124 (2013). 10.1104/pp.113.221150 PMC3729786 23784463

[56] Agrawal, A. A. Current trends in the evolutionary ecology of plant defence. *Funct. Ecol.* **25**, 420–432 (2011).

[57] Sinclair, F. H. et al. Impacts of local adaptation of forest trees on associations with herbivorous insects: implications for adaptive forest management. *Evol. Appl.* **8**, 972–987 (2015). 10.1111/eva.12329 PMC4662346 26640522

[58] Caffarra, A. & Donnelly, A. The ecological significance of phenology in four different tree species: effects of light and temperature on bud burst. *Int. J. Biometeorol.* **55**, 711–721 (2011). 10.1007/s00484-010-0386-1 21113629

[59] Chesson, P. Mechanisms of maintenance of species diversity. *Ann. Rev. Ecol. Evol. Syst.* **31**, 343–366 (2000).

[60] Tilman, D. *Resource Competition and Community Structure* (Princeton Univ. Press, 1982). 7162524

[61] Tuomi, J., Niemelä, P., Jussila, I., Vuorisalo, T. & Jormalainen, V. Delayed budbreak: a defensive response of mountain birch to early-season defoliation?. *Oikos* **54**, 87–91 (1989).

[62] Varley, G. C., Gradwell, G. R. & Hassell, M. P. *Insect Population Ecology: An Analytical Approach* (Blackwell Scientific, 1973).

[63] Myers, J. H. & Cory, J. S. Population cycles in forest Lepidoptera revisited. *Annu. Rev. Ecol. Evol. Syst.* **44**, 565–592 (2013).

[64] Haukioja, E. On the role of plant defences in the fluctuation of herbivore populations. *Oikos* **35**, 202–213 (1980).

[65] Neuvonen, S., Haukioja, E. & Molarius, A. Delayed inducible resistance against a leaf-chewing insect in four deciduous tree species. *Oecologia* **74**, 363–369 (1987). 10.1007/BF00378931 28312474

[66] Frison, P.-L. et al. Potential of Sentinel-1 data for monitoring temperate mixed forest phenology. *Remote Sens.* **10**, 2049 (2018).

[67] Dixon, D. J., Zhu, Y. & Jin, Y. Canopy height estimation from PlanetScope time series with spatio-temporal deep learning. *Remote Sens. Environ.* **318**, 114518 (2025).

[68] Wood, S. N. Fast stable restricted maximum likelihood and marginal likelihood estimation of semiparametric generalized linear models. *J. R. Stat. Soc. B* **73**, 3–36 (2011).

[69] R Core Team. *R: A Language and Environment for Statistical Computing* (R Foundation for Statistical Computing, 2025).

[70] Mallick, S., Bae, S., Lichter, J. & Müller, J. Forest trees delay budburst across landscapes to escape herbivory—data and code. *Zenodo*10.5281/zenodo.17285429 (2026). PMC13345905 42067638

