# Supplementary material for: Satellite data show trees delay budburst across landscapes to escape herbivores
Source: Nat Ecol Evol. 2026 May 1;10(7):1287–95. doi: 10.1038/s41559-026-03071-9 (PMC13345905; doi:10.1038/s41559-026-03071-9)
Supplement: Supplementary file 1 — Supplementary Video caption, Methods 1–3 and References. [file 41559_2026_3071_MOESM1_ESM.pdf]

nature ecology & evolution

Supplementary information https://doi.org/10.1038/s41559-026-03071-9

Satellite data show trees delay budburst across landscapes to escape herbivores

In the format provided by the authors and unedited

Table of contents

Page

Supplementary Video 1:
Interannual shift in budburst phenology 2

Supplementary Methods 1:
Leaf herbivory leads to budburst delay irrespective of changes in physiological status of trees due to herbivory 3

Supplementary Methods 2:
Interpretation of budburst delay and herbivory reduction in common units 4

Supplementary Methods 3:
Budburst delay reduces folivory irrespective of folivore control by natural antagonists 5

References 7

Supplementary Video 1: Interannual shift in budburst phenology.

This video illustrates year-to-year shifts in budburst timing among trees in the four treatment plots (HC, HI, LC, and LI) within Block A. Satellite-detected pixels are colour-coded based on relative budburst timing: white (late) → green (early). The study year is indicated in the top-left corner of each frame.

Supplementary Methods 1: Leaf herbivory leads to budburst delay irrespective of changes in physiological status of trees due to herbivory

We found that prior leaf herbivory was associated with delayed budburst (Fig. 2a). Herbivory reduces the resource levels in trees, which may in turn delay the breaking of bud dormancy and thus delay budburst. Such a byproduct of low resource levels would not be an adaptation in response to natural selection, even if it happens to be beneficial in reducing future herbivory. Adaptation becomes more plausible as an interpretation if the relationship between herbivory and budburst delays persists after accounting for resource level of trees. A frequently used proxy of resource level is stem growth[1,2]. Data on stem growth were available, for 44 out of the 60 sites and 3 years (2019 and 2021): every year in the fall season we measured the diameter at breast height in mm for 20 oak trees per site (from site centre, 5 trees along East, West, South and North axes). For each tree between two consecutive years, we calculated relative growth rate by the formula: $(DBH_{y=n+1} - DBH_{y=n})/((DBH_{y=n+1} + DBH_{y=n})/2)$. Because these data could not be matched directly to satellite pixels, we averaged growth rates at the site level. Including these site-level covariates, we repeated the analyses corresponding to Fig. 2a in the main text. After accounting for tree growth rates, trees with higher prior herbivory still showed significantly delayed budburst ($n = 21,787$, $F = 125.4$, $p < 0.001$, adjusted $R^2 = 0.095$). Tree growth rate also contributed significantly ($F = 4.9$, $p = 0.001$), though its effect size was an order of magnitude lower than that of prior herbivory ($F = 125.4$ vs. 4.9).

Supplementary Methods 2: Interpretation of budburst delay and herbivory reduction in common units

Satellite monitoring allows for broad spatial and temporal coverage of budburst and herbivory, and the resulting data are in the form of unitless vegetation indices. While these indices effectively capture relative differences across trees and sites, they are not easily interpretable in conventional units such as days or percentage leaf loss. To facilitate intuitive interpretation, we applied additional post-processing steps to estimate average shifts in budburst timing (in days) and reductions in herbivory (in percentage). These estimates involve several approximations and are intended solely for summarising overall trends; all statistical analyses were conducted using the original, more accurate index values of canopy development.

Estimating changes in budburst timing (in days): For each year, we first calculated the mean Normalized Canopy Development Index (NCDI) across 27,500 satellite-detected pixels at the midpoint of the green-up period. This across-pixel mean NCDI value was used as the reference value corresponding to budburst for that year. For each pixel, we then identified the day of year (DOY) when its NCDI value first reached this budburst threshold. To compare budburst timing across years, we mean-centred the DOY values within each year to zero. For each pixel, the annual shift in budburst timing (in days) was calculated as the difference in mean-centred DOY values between consecutive years. Finally, we grouped trees each year according to whether they experienced below- or above-median herbivory (see Methods for details on herbivory estimation) and computed the mean difference in annual shift in budburst timing (in days) between these two groups.

Estimating changes in herbivory (in percentages): For each pixel, we calculated the year-to-year percentage change in herbivory by dividing the ‘change in herbivory index’ by the ‘herbivory index’ from the earlier year (details of these indices are given in Online Methods), then multiplying by 100. From these, we selected the subset of pixels that showed a delay in budburst from one year to the next. We then averaged the percentage changes in herbivory across all delayed pixels to estimate the average herbivory reduction associated with budburst delay.

Supplementary Methods 3: Budburst delay reduces folivory irrespective of folivore control by natural antagonists or of abiotic conditions

We found that delayed budburst between years was associated with reduced folivory (Fig. 2d), and this association was stronger in forest sites experiencing folivore outbreaks (Fig. 2f). However, significant associations alone do not establish direct causality. Trees subject to heavy folivory are also regulated by natural antagonists such as parasitoids and pathogens (baculovirus). These biotic controls typically act with a delayed density-dependent response: parasitoids and viruses often reduce folivore populations in the following year rather than immediately. Consequently, the observed reduced folivory could simply result from delayed response by natural antagonists to high folivore activity in the previous year. To disentangle these effects and test the robustness of our results, it was necessary to account for such biotic controls.

Our main analyses were conducted at the scale of satellite pixels across 60 forest sites from 2017 to 2021. Whereas our data on parasitoids[3] and viral infections[4] could only be available at the site level, and for a smaller subset: 48 sites and 2–3 years, depending on the enemy group. Between 2019 and 2021, we collected a total of 20,299 caterpillars from 48 sites using canopy fogging. Caterpillars were individually screened for parasitoid infection via metabarcoding, complemented with visual identification of exoparasitoids. For viral infections, in 2019 and 2020 we randomly selected 10 *Lymantria dispar* caterpillars per site each year and diagnosed infection by the baculovirus Lymantria dispar multiple nucleopolyhedrovirus (LdMNPV). Because these data could not be matched directly to satellite pixels, we averaged parasitism and infection rates at the site level. Including these site-level covariates, we repeated the analyses corresponding to Fig. 2d and 2f in the main text.

After accounting for parasitism and viral infection rates, trees with delayed budburst still showed significantly reduced folivory ($n = 23{,}730$, $F = 562.3$, $p < 0.001$, adjusted $R^2 = 0.218$). Parasitism rate also contributed significantly ($F = 9.9$, $p = 0.002$), though its effect size was an order of magnitude lower than that of budburst delay ($F = 562.3$ vs. 9.9). Viral infection rate did not significantly influence folivory.

When restricting the analyses to the outbreak year 2019, delayed budburst remained the main predictor of folivory reduction ($n = 14{,}291$, $t = 27.9$, $p < 0.001$, adjusted $R^2 = 0.234$); both parasitism rate ($t = 11.4$, $p < 0.001$) and viral infection rate ($t = 12.6$, $p < 0.001$) had significant but comparatively minor contributions. Most importantly, accounting for the effects of parasitism rate and viral infection rate did not alter the main result: the reduction of folivory by budburst delay remained greater under outbreak conditions ($t = 2.6$, $p = 0.009$).

Similar to the possible effect of the natural antagonists, there might also be confounding effects of the abiotic factors, which can be captured well by Ellenberg indicator values (light, temperature, continentality, moisture, reaction and nutrient availability) extracted from herb layer composition[5]. These semi-quantitative indices characterize local environmental conditions and are widely applied as proxies for long-term abiotic conditions and have been shown to reliably capture site differences in resource availability and microclimate[6]. Importantly, integrating year-round environmental conditions across our 60 study sites using direct measurements would require permanent, long-term monitoring infrastructure, making these indicator values one of the few practical alternatives. Hence, in a parallel analysis we added these values as covariates.

After accounting for Ellenberg indicator values, trees with delayed budburst still showed significantly reduced folivory ($n = 93{,}872$, $F = 1219.5$, $p < 0.001$, adjusted $R^2 = 0.144$). Where none of these indicators for local site conditions influenced folivory.

The pixel-level analyses given above were also conducted at the site level. Site-level patterns of adjusted R^2 remained robust (in every case $p < 0.001$, $t = 4.513$-11.951) even after controlling for potential effects of abiotic conditions (as inferred from indicator values of herbaceous vegetation). Furthermore, the analyses had higher adjusted R^2 (0.548) when none of these confounding factors were included, then when either of the possible confounders were accounted for (0.347 to 0.480).

In this context, we note that despite 2018 being the drought year in the region, it was not the year with highest herbivory. In 2019, the outbreak year, trees had significantly higher herbivory than in 2018 ($t = -11.503$, $p < 0.001$). Moreover, herbivory level in 2018 did not differ from 2020, a regular year without any particularity such as drought or herbivore outbreak ($t = -1.246$, $p = 0.598$).

References:

1. Wang, T. *et al.* Dynamics of forest net primary productivity based on tree ring reconstruction in the Tianshan Mountains. *Ecological Indicators* **146**, 109713 (2023).

2. Pretzsch, H. Trees grow modulated by the ecological memory of their past growth. Consequences for monitoring, modelling, and silvicultural treatment. *Forest Ecology and Management* **487**, 118982 (2021).

3. Wolz, M. *et al.* Response of parasitoid communities to insecticide application during a Lymantria dispar outbreak in mixed oak forests. *Journal of Applied Ecology* **61**, 2774–2785 (2024).

4. Oehlmann, C. *et al.* Amplicon-based analyses of single nucleotide polymorphisms reveal the genetic structure of a forest insect baculovirus. *Virus Evol* veaf061 (2025) doi:10.1093/ve/veaf061.

5. Ewald, J. 2003. The sensitivity of Ellenberg indicator values to the completeness of vegetation releves. *Basic and Applied Ecology* **4**:507-513.

6. Diekmann, M. Species indicator values as an important tool in applied plant ecology – a review. *Basic and Applied Ecology* **4**, 493–506 (2003).
