# Supplementary material for: Satellite data show trees delay budburst across landscapes to escape herbivores
Source: Nat Ecol Evol. 2026 May 1;10(7):1287–95. doi: 10.1038/s41559-026-03071-9 (PMC13345905; doi:10.1038/s41559-026-03071-9)
Supplement: Supplementary file 2 — Reporting Summary [file 41559_2026_3071_MOESM2_ESM.pdf]

nature portfolio

Corresponding author(s): Soumen Mallick

Last updated by author(s): Mar 10, 2026

Reporting Summary

Nature Portfolio wishes to improve the reproducibility of the work that we publish. This form provides structure for consistency and transparency in reporting. For further information on Nature Portfolio policies, see our Editorial Policies and the Editorial Policy Checklist.

Statistics

For all statistical analyses, confirm that the following items are present in the figure legend, table legend, main text, or Methods section.

n/a	Confirmed	
☐	☒	The exact sample size (*n*) for each experimental group/condition, given as a discrete number and unit of measurement
☐	☒	A statement on whether measurements were taken from distinct samples or whether the same sample was measured repeatedly
☐	☒	The statistical test(s) used AND whether they are one- or two-sided<br>*Only common tests should be described solely by name; describe more complex techniques in the Methods section.*
☐	☒	A description of all covariates tested
☒	☐	A description of any assumptions or corrections, such as tests of normality and adjustment for multiple comparisons
☐	☒	A full description of the statistical parameters including central tendency (e.g. means) or other basic estimates (e.g. regression coefficient) AND variation (e.g. standard deviation) or associated estimates of uncertainty (e.g. confidence intervals)
☐	☒	For null hypothesis testing, the test statistic (e.g. *F*, *t*, *r*) with confidence intervals, effect sizes, degrees of freedom and *P* value noted<br>*Give P values as exact values whenever suitable.*
☒	☐	For Bayesian analysis, information on the choice of priors and Markov chain Monte Carlo settings
☐	☒	For hierarchical and complex designs, identification of the appropriate level for tests and full reporting of outcomes
☐	☒	Estimates of effect sizes (e.g. Cohen's *d*, Pearson's *r*), indicating how they were calculated

Our web collection on statistics for biologists contains articles on many of the points above.

Software and code

Policy information about availability of computer code

Data collection	Microsoft® Excel for Mac Version 16.102.3<br>Sentinel-1 Toolbox of Sentinel Application Platform (SNAP) Version 7.0
Data analysis	R Version 4.5.1 and 4.2.1<br>RStudio Version 2025.09.1+401

For manuscripts utilizing custom algorithms or software that are central to the research but not yet described in published literature, software must be made available to editors and reviewers. We strongly encourage code deposition in a community repository (e.g. GitHub). See the Nature Portfolio guidelines for submitting code & software for further information.

Data

Policy information about availability of data

All manuscripts must include a data availability statement. This statement should provide the following information, where applicable:

- Accession codes, unique identifiers, or web links for publicly available datasets
- A description of any restrictions on data availability
- For clinical datasets or third party data, please ensure that the statement adheres to our policy

The data are publicly available on Zenodo: https://doi.org/10.5281/zenodo.17285429
The R code is publicly available on Zenodo: https://doi.org/10.5281/zenodo.17285429

Research involving human participants, their data, or biological material

Policy information about studies with human participants or human data. See also policy information about sex, gender (identity/presentation), and sexual orientation and race, ethnicity and racism.

Reporting on sex and gender	*Use the terms sex (biological attribute) and gender (shaped by social and cultural circumstances) carefully in order to avoid confusing both terms. Indicate if findings apply to only one sex or gender; describe whether sex and gender were considered in study design; whether sex and/or gender was determined based on self-reporting or assigned and methods used. Provide in the source data disaggregated sex and gender data, where this information has been collected, and if consent has been obtained for sharing of individual-level data; provide overall numbers in this Reporting Summary. Please state if this information has not been collected. Report sex- and gender-based analyses where performed, justify reasons for lack of sex- and gender-based analysis.*
Reporting on race, ethnicity, or other socially relevant groupings	*Please specify the socially constructed or socially relevant categorization variable(s) used in your manuscript and explain why they were used. Please note that such variables should not be used as proxies for other socially constructed/relevant variables (for example, race or ethnicity should not be used as a proxy for socioeconomic status). Provide clear definitions of the relevant terms used, how they were provided (by the participants/respondents, the researchers, or third parties), and the method(s) used to classify people into the different categories (e.g. self-report, census or administrative data, social media data, etc.) Please provide details about how you controlled for confounding variables in your analyses.*
Population characteristics	*Describe the covariate-relevant population characteristics of the human research participants (e.g. age, genotypic information, past and current diagnosis and treatment categories). If you filled out the behavioural & social sciences study design questions and have nothing to add here, write "See above."*
Recruitment	*Describe how participants were recruited. Outline any potential self-selection bias or other biases that may be present and how these are likely to impact results.*
Ethics oversight	*Identify the organization(s) that approved the study protocol.*

Note that full information on the approval of the study protocol must also be provided in the manuscript.

Field-specific reporting

Please select the one below that is the best fit for your research. If you are not sure, read the appropriate sections before making your selection.

☐ Life sciences ☐ Behavioural & social sciences ☒ Ecological, evolutionary & environmental sciences

For a reference copy of the document with all sections, see nature.com/documents/nr-reporting-summary-flat.pdf

Ecological, evolutionary & environmental sciences study design

All studies must disclose on these points even when the disclosure is negative.

Study description	Monitoring the budburst phenology and leaf herbivory of 27,500 satellite-detected pixels (each approximately corresponding to an individual tree crown) across 60 forest sites and for 5 years.
Research sample	10 x 10 m pixels, each primarily corresponding to an individual tree crown.
Sampling strategy	High-resolution satellite radar data.
Data collection	Soyeon Bae obtained Sentinel-1 C-band SAR data covering our study area from the ESA Scientific Hub (https://scihub.copernicus.eu/). All available level-1 groundrange-detected high-resolution (GRDH) products acquired by the interferometric wide-swath mode were selected, including from both ascending (relative orbits of 117 and 15) and descending (relative orbits of 168 and 66) satellite passes. These were pre-processed using the Sentinel Application Platforms (SNAP) Sentinel-1 Toolbox software and then Normalized Canopy Development Indices were calculated (full details in DOI: 10.1111/2041-210X.13726). These indices were used to calculate budburst phenology and leaf herbivory as described in the Methods.
Timing and spatial scale	Timing: from 2017 to 2021 (March to September every year)<br>Spatial scale: 60 forest sites spanning over 2400 km² in Franconia, Bavaria, Germany (as shown in Extended Data Fig. 1).
Data exclusions	No data were excluded from the study.
Reproducibility	The conclusions are drawn from patterns observed over 5 years and across 27,500 trees spreading over 60 forest sites spanning approximately 2400 km².
Randomization	No randomization is required as we monitored ALL the 'trees' (i.e. satellite-detected pixels) from our 60 forest sites.
Blinding	No blinding is required as we monitored ALL the 'trees' (i.e. satellite-detected pixels) from our 60 forest sites.

Did the study involve field work? ☒ Yes ☐ No

Field work, collection and transport

Field conditions	The study area has a temperate climate, with mean annual temperatures ranging from 7.5°C to 9.0°C and annual precipitation between 600 mm and 1000 mm.
Location	60 forest sites spanning over 2400 km² in Franconia, Bavaria, Germany (200–500 m a.s.l., centered at N 49°37', E 10°24', as shown in Extended Data Fig. 1).
Access & import/export	The fieldwork did not involve import/export of samples. In 2019, the outbreak year, we arialy applied insecticide in half of the forest sites (as described in the Methods).
Disturbance	No disturbance caused.

Reporting for specific materials, systems and methods

We require information from authors about some types of materials, experimental systems and methods used in many studies. Here, indicate whether each material, system or method listed is relevant to your study. If you are not sure if a list item applies to your research, read the appropriate section before selecting a response.

Materials & experimental systems

n/a	Involved in the study	
☒	☐	Antibodies
☒	☐	Eukaryotic cell lines
☒	☐	Palaeontology and archaeology
☒	☐	Animals and other organisms
☒	☐	Clinical data
☒	☐	Dual use research of concern
☐	☒	Plants

Methods

n/a	Involved in the study	
☒	☐	ChIP-seq
☒	☐	Flow cytometry
☒	☐	MRI-based neuroimaging

Plants

Seed stocks	No seed stock used. We monitored trees in forests using satellite remote sensing.
Novel plant genotypes	No plant genotype information is available. We monitored trees in forests using satellite remote sensing.
Authentication	Authentication not required as no seed stock was used.
