# Supplementary material for: Satellite data show trees delay budburst across landscapes to escape herbivores
Source: Nat Ecol Evol. 2026 May 1;10(7):1287–95. doi: 10.1038/s41559-026-03071-9 (PMC13345905; doi:10.1038/s41559-026-03071-9)
Supplement: Supplementary file 3 — Peer Review File [file 41559_2026_3071_MOESM3_ESM.pdf]

nature portfolio

Peer Review File

Satellite data show trees delay budburst across landscapes to escape herbivores

Corresponding Author: Dr Soumen Mallick

This file contains all editorial decision letters in order by version, followed by all author rebuttals in order by version.

Version 0:

Reviewer comments:

Reviewer #1

(Remarks to the Author)
In this study, the authors claim the finding that prior-year herbivory delays budburst of next year, and that such delay can substantially reduce herbivory damage (55%) in that next year. They suggest this to be an adaptive plastic response. The study was based on spatial-temporal analysis of radar data (Sentinel 1) for 60 oak forests (each of ca. 4.6 ha on average) in Southern Germany (some of which comprising ancillary data of insecticide application and/or herbivores natural antagonists). I must say I'm not fully convinced by the study, but I'm open to be corrected, if I misunderstood some points. My main reservations are listed below.

1.before any inference and data analysis, these is the need to be sure about the accuracy of the budburst and defoliation data. The authors should explain (shortly, in main text) and in more details (in methods), the method of Bae at al 2022, which is at the foundation of their study (I find the details currently available too limited). To have information and insights on the methods, I had to go to the original paper of Bae at al 2022. So, I have realized that in the original Bae et al. study, (i) the analysis was done at plot level (4.6 ha) and not at the tree level, (ii) that the (very important) validation (with TLS data) was based on area of ca. 0.5 ha and that (iii) the index they developed (and fundamental in this study as shown in Extended Data Fig 3) is based on 12 days aggregated point. Based on this, you should convince the reader that your data at 'tree level' and detecting the short interval of '3 days' delay are robust, considering also all biases with radar assessment (also mentioned in Bae et al). My knowledge is limited, but I know few tree level RS assessments of phenology, one of which Eitel at al 2023 (https://doi.org/10.1016/j.agrformet.2023.109549), using much higher resolution data (PlanetScope, 3 m and 1 day). Also your statement that "27.500 10 x 10 pixels, each primarily corresponding to an individual tree crown" should be substantiated, as, for this statement, you would need tree density, location detected by radar and crown size, all matching, which personally I find it difficult. Overall, if there is uncertainty in the budburst/defoliation data and the index used (see Extended Data Fig 3), I would argue that only a combination of these data with a controlled manipulative experiment on herbivores could give a clear answer to your questions.
2.the most accurate projections that we can have today about budburst timing are based on (though incomplete) understanding and simulation of bud dormancy (see e.g. review 10.3389/fphgy.2023.1293057). How do you see your hypothesized conclusions (herbivory delaying next year budburst), fitting into this in term of processes and mechanisms? Would it not be, more simply, possible that the delay you see after herbivory and defoliation, is a physiological response only triggered by a weakened tree status (by herbivory) on next year phenology e.g. lower nutrients and C reserves? (see the advance in budburst related to nutrient status as described by doi.org/10.3389/fpls.2016.01641and doi:10.1093/jxb/ert061, implicitly indicating later budburst in case of lower nutrients). Thus an interannual physiological response (legacy effect) rather than an adaptation? In this context, had drought (e.g. in 2018) any impact on defoliation? Can you exclude any confounding effect with abiotic factors? (as you accurately have done for biotic factors in Supplementary Methods 2).
3.in figure Extended Data Fig 3, it is clear that the herbivory period in the region in quite wide (> 60 days). Can you provide additional substantiation that only a 3 days shift would be so crucial in reducing so much herbivory damage (55%!)? Any experimental evidence showing similar results?
4. a more general comment: assuming that your conclusions are correct, should we not expect also a shift (delay) in phenology of herbivores too, following the delays of tree budburst? (in comparison than trees, their lifetime is so short and new generation so fast, that we can expect to be more adaptive in the long-term…..)

Reviewer #2

(Remarks to the Author)
This study examines the interactive effects and underlying drivers of budburst timing on herbivory in central European deciduous forests. The studies combines very impressive experimental manipulations across a large network of forests, with a novel series of remote sensing analyses. The results are highly novel and very convincing for the most part, they are highly relevant to anyone interested in understanding plant dynamics. The manuscript is generally well written though there are multiple, fairly minor areas which are rather unclear, and the analyses seem appropriate for the most part. I have two larger issues:

(1) The logic and context underlying the study hypotheses/expectations need to be more clearly outlined in the introduction. This is particularly the case for questions 1 and 2, in particular the rationale for the expectations for why the responses in each case should be graded with a weaker response at outbreak sites. Then the results need to be more clearly tied to these questions. At several points in the results & discussion there are mentions of expectations and hypotheses and it's not clear if/how these overlap with the questions, and if so which specific question.
(2) I'm not clear that the analysis underlying fig 3 acheives what the authors intend. To compare the magnitude of two responses (budburst delay in response to herbivory & herbivory suppression in response to budburst delay) surely you should correlate the slopes of these two relationships? The r2 will tend to be positively correlated to the slopes, but captures rather different information to what you want to address your question I think

See below for minor comments:

Line 60) I like this observation. What is missing is some mention beforehand that herbivory itself may not be a static factor but may change directionally with climate change and other factors.
Lines 56-58) This sentence felt like it should be the final conclusion.
Lines 65-66) Some brief review with relevant references would be useful here of other possible drivers of budburst, if there are any, besides climate change and herbivory.
Lines 68-69) Rephrase "may erode tree responses", doesn't quite make sense at present and is rather vague, what responses, erode how/in what direction?
Lines 74-75) Before this statement you should first clarify with evidence that spring is the peak time for herbivory. Also, can you give some assessment of what portion of the overall herbivore community are "spring-feeders".
Lines 84-85) This point is not clear to me, why does the fact that outbreaks last only a year mean that they are a poor proxy?
Lines 98-101) This first sentence of the paragraph seems unnecessary.
Lines 114-115) I don't understand the last part of question 2, why would the response be weaker at outbreak sites? The background context and logic for the questions needs to be more clearly described in the introduction.
Lines 161-162) This is particularly important if there may be reasons to suggest that insect populations and herbivory could be consistently changing. See references below.
https://royalsocietypublishing.org/rsbl/article/11/7/20150480/50718/Changes-in-the-background-losses-of-woody-plant
https://www.science.org/doi/10.1126/science.aax9931
https://www.nature.com/articles/s41586-023-06861-4
But see: https://onlinelibrary.wiley.com/doi/10.1111/gcb.70587
Lines 164-165) This last part doesn't make sense to me, why would this provide a less informative signal?
Lines 165-169) Isn't one important aspect missed here the relative timing of herbivory in the season, or the proportion of annual herbivory at a site which is concentrated in the spring. There will likely be substantial variation in this aspect with important implications for budburst, sites/tree which have much more herbivory in the spring should benefit much more from delayed budburst than sites/trees where herbivory is more evenly distributed through the summer, or that occurs later.
Line 169) This expectation needs to be clearly described earlier in the introduction, preferably explicitly linked to one of the 3 study questions. I'm not sure why you would expect this, wouldn't you expect that the more severely attacked sites, there would be relatively greater benefit in delaying budburst.
Figure 2) "Weakest" is currently misspelled. Some more clarification in the text and/or caption is needed about what you mean by the strongest and weakest site-years.
Lines 196) Related how? Always give specific magnitude/direction of relationship if possible/appropriate, this is much more informative
Line 200) "before-year" isn't really used though it makes good sense, more usual would be to say "prior/previous year"
Lines 200-201) Not clear what you mean by "between-herbivore competition" which of the two factors mentioned does this apply to, or does it apply to both?
Lines 208-209) This is a remarkable result. Here you give some estimate of the mean/range in delays encountered. Given that the delays quite short (just a few days), this suggests that in this system/moth a huge portion of the annual herbivory occurs in just a few days.
Lines 222-223) This makes it sound like somehow delayed budburst causes more rapid maturation, whereas I guess they are just associated together. Plants that delay budburst are under greater pressure to "catchup". If delaying budburst is such a profitable strategy, and if any delays can be compensated for by more rapid development…why don't all trees use this strategy?
Lines 227-228) This expectation needs to be better explained/justified in the introduction.
Lines 230-232) Again, this expectation was not clearly described/motivated earlier in the introduction. Also, can you describe in words what this interaction means, to help with interpretation.
Lines 234-235) Its not clear to me why this result challenges this assumption. The interaction you observed doesn't mean that trees in the outbreak weren't "overwhelmed" whatever you mean by this, it just means that their strategy the next year was different. Am I misunderstanding something?
Line 240) This should be "predicting", also you can remove the "a" at the end of this line.
Lines 256-258) Fascinating result. But I'm a bit confused, if you're interested in the magnitude of the response (of budburst

and herbivory reduction) shouldn't you use the slope of the relationships, not their r2? The r2 of a strong relationship (greater response of Y to a change in X) can still be lower than a relatively weak relationship. Similarly, you can easily of course have two sites with the same relationship between Y and X but very different r2 values. Though generally there will tend to be a positive correlation between slope and r2
Lines 293-296) Very interesting discussion. Somewhere perhaps here you need to acknowledge the extent to which your conclusions may be limited by the particularities of the herbivore species studied. Also, are there any rough estimates of what portion of the herbivore communities have narrow vs broad temporal niches. For this to have the major, broadly applicable implications you are seeking to make there needs to be a sizeable component of many herbivore communities which have a narrow temporal niche….is this the case?
Line 311) Should be "complement to the".
Line 313) I suggest you delete "major", it's quite vague, subjective in this case.
Lines 314) Effective yes, widespread is a stretch I think. You demonstrate this over a large area but still within oak forests in central Europe. Some extra discussion on differences/similarities between your study system (including herbivore communities) and others, would help the reader to judge how widespread these results are likely to be.
Lines 317-322) OK, now you make this case, good. This should come already in the introduction, preferably with references supporting your claims.
Lines 331) You never really clearly made the point in the introduction that anthropogenic activity might have an effect of the spatial and temporal pattern of herbivory.

Decision Letter:

4th December 2025

*Please ensure you delete the link to your author homepage in this e-mail if you wish to forward it to your co-authors.

Dear Soumen,

Your manuscript entitled "Satellite data show trees delay budburst across landscapes to escape herbivores" has now been assessed by two reviewers. As you will see from the reports below, the reviewers find the work of potential interest but have raised a number of concerns which cast doubt on the strength of the conclusions. We would therefore like to see your responses to the criticisms raised and a revised manuscript to decide on whether the study is publishable in Nature Ecology & Evolution.

When revising your manuscript:

* Please provide the manuscript in Microsoft Word format and highlight all changes in the same file or a copy.

* Include a "Response to reviewers" document detailing, point-by-point, how you addressed each reviewer comment. If no action was taken to address a point, you must provide a compelling argument. This response will be sent back to the reviewers along with the revised manuscript.

* If you have not done so already please begin to revise your manuscript so that it conforms to our Article format instructions at http://www.nature.com/natecolevol/info/final-submission. Refer also to any guidelines provided in this letter.

* Include a revised version of any required reporting checklist. It will be available to referees (and, potentially, statisticians) to aid in their evaluation if the manuscript goes back for peer review. A revised checklist is essential for re-review of the paper.

* Extended Data Figures - please ensure that any supplementary figures and tables that are crucial to the manuscript's conclusions are converted into Extended Data figures and tables to increase visibility of these data. Extended Data figures and tables are online-only (present in the online PDF and full-text HTML versions of the paper), peer-reviewed display items that provide essential background to the article but are not included in the main article due to space constraints. A maximum of ten Extended Data display items (figures and tables) is permitted.

We hope to receive your revised manuscript within three months. If you cannot send it within this time, please let us know. We will be happy to consider your revision so long as nothing similar has been accepted for publication at Nature Ecology & Evolution or published elsewhere. Once ready, please use the link below to submit your revised manuscript and related files:

Link Redacted

Note: This URL links to your confidential home page and associated information about manuscripts you may have submitted, or that you are reviewing for us. If you wish to forward this email to co-authors, please delete the link to your homepage.

Nature Ecology & Evolution is committed to improving transparency in authorship. As part of our efforts in this direction, we are now requesting that all authors identified as 'corresponding author' on published papers create and link their Open Researcher and Contributor Identifier (ORCID) with their account on the Manuscript Tracking System (MTS), prior to acceptance. ORCID helps the scientific community achieve unambiguous attribution of all scholarly contributions. You can

create and link your ORCID from the home page of the MTS by clicking on ‘Modify my Springer Nature account’. For more information please visit please visit www.springernature.com/orcid.

Please do not hesitate to contact me if you have any questions or would like to discuss these revisions further.

Best wishes,
[Redacted]

****

Reviewers' comments:

Reviewer #1 (Remarks to the Author):

In this study, the authors claim the finding that prior-year herbivory delays budburst of next year, and that such delay can substantially reduce herbivory damage (55%) in that next year. They suggest this to be an adaptive plastic response. The study was based on spatial-temporal analysis of radar data (Sentinel 1) for 60 oak forests (each of ca. 4.6 ha on average) in Southern Germany (some of which comprising ancillary data of insecticide application and/or herbivores natural antagonists). I must say I’m not fully convinced by the study, but I’m open to be corrected, if I misunderstood some points. My main reservations are listed below.

1.before any inference and data analysis, these is the need to be sure about the accuracy of the budburst and defoliation data. The authors should explain (shortly, in main text) and in more details (in methods), the method of Bae at al 2022, which is at the foundation of their study (I find the details currently available too limited). To have information and insights on the methods, I had to go to the original paper of Bae at al 2022. So, I have realized that in the original Bae et al. study, (i) the analysis was done at plot level (4.6 ha) and not at the tree level, (ii) that the (very important) validation (with TLS data) was based on area of ca. 0.5 ha and that (iii) the index they developed (and fundamental in this study as shown in Extended Data Fig 3) is based on 12 days aggregated point. Based on this, you should convince the reader that your data at ‘tree level’ and detecting the short interval of ‘3 days’ delay are robust, considering also all biases with radar assessment (also mentioned in Bae et al). My knowledge is limited, but I know few tree level RS assessments of phenology, one of which Eitel at al 2023 (https://doi.org/10.1016/j.agrformet.2023.109549), using much higher resolution data (PlanetScope, 3 m and 1 day). Also your statement that “27.500 10 x 10 pixels, each primarily corresponding to an individual tree crown” should be substantiated, as, for this statement, you would need tree density, location detected by radar and crown size, all matching, which personally I find it difficult. Overall, if there is uncertainty in the budburst/defoliation data and the index used (see Extended Data Fig 3), I would argue that only a combination of these data with a controlled manipulative experiment on herbivores could give a clear answer to your questions.
2.the most accurate projections that we can have today about budburst timing are based on (though incomplete) understanding and simulation of bud dormancy (see e.g. review 10.3389/fphgy.2023.1293057). How do you see your hypothesized conclusions (herbivory delaying next year budburst), fitting into this in term of processes and mechanisms? Would it not be, more simply, possible that the delay you see after herbivory and defoliation, is a physiological response only triggered by a weakened tree status (by herbivory) on next year phenology e.g. lower nutrients and C reserves? (see the advance in budburst related to nutrient status as described by doi.org/10.3389/fpls.2016.01641and doi:10.1093/jxb/ert061, implicitly indicating later budburst in case of lower nutrients). Thus an interannual physiological response (legacy effect) rather than an adaptation? In this context, had drought (e.g. in 2018) any impact on defoliation? Can you exclude any confounding effect with abiotic factors? (as you accurately have done for biotic factors in Supplementary Methods 2).
3.in figure Extended Data Fig 3, it is clear that the herbivory period in the region in quite wide (> 60 days). Can you provide additional substantiation that only a 3 days shift would be so crucial in reducing so much herbivory damage (55%!)? Any experimental evidence showing similar results?
4. a more general comment: assuming that your conclusions are correct, should we not expect also a shift (delay) in phenology of herbivores too, following the delays of tree budburst? (in comparison than trees, their lifetime is so short and new generation so fast, that we can expect to be more adaptive in the long-term…..)

Reviewer #2 (Remarks to the Author):

This study examines the interactive effects and underlying drivers of budburst timing on herbivory in central European deciduous forests. The studies combines very impressive experimental manipulations across a large network of forests, with a novel series of remote sensing analyses. The results are highly novel and very convincing for the most part, they are highly relevant to anyone interested in understanding plant dynamics. The manuscript is generally well written though there are multiple, fairly minor areas which are rather unclear, and the analyses seem appropriate for the most part. I have two larger issues:

(1) The logic and context underlying the study hypotheses/expectations need to be more clearly outlined in the introduction. This is particularly the case for questions 1 and 2, in particular the rationale for the expectations for why the responses in each case should be graded with a weaker response at outbreak sites. Then the results need to be more clearly tied to these questions. At several points in the results & discussion there are mentions of expectations and hypotheses and it’s not clear if/how these overlap with the questions, and if so which specific question.

(2) I'm not clear that the analysis underlying fig 3 acheives what the authors intend. To compare the magnitude of two responses (budburst delay in response to herbivory & herbivory suppression in response to budburst delay) surely you should correlate the slopes of these two relationships? The r2 will tend to be positively correlated to the slopes, but captures rather different information to what you want to address your question I think

See below for minor comments:

Line 60) I like this observation. What is missing is some mention beforehand that herbivory itself may not be a static factor but may change directionally with climate change and other factors.
Lines 56-58) This sentence felt like it should be the final conclusion.
Lines 65-66) Some brief review with relevant references would be useful here of other possible drivers of budburst, if there are any, besides climate change and herbivory.
Lines 68-69) Rephrase "may erode tree responses", doesn't quite make sense at present and is rather vague, what responses, erode how/in what direction?
Lines 74-75) Before this statement you should first clarify with evidence that spring is the peak time for herbivory. Also, can you give some assessment of what portion of the overall herbivore community are "spring-feeders".
Lines 84-85) This point is not clear to me, why does the fact that outbreaks last only a year mean that they are a poor proxy?
Lines 98-101) This first sentence of the paragraph seems unnecessary.
Lines 114-115) I don't understand the last part of question 2, why would the response be weaker at outbreak sites? The background context and logic for the questions needs to be more clearly described in the introduction.
Lines 161-162) This is particularly important if there may be reasons to suggest that insect populations and herbivory could be consistently changing. See references below.
https://royalsocietypublishing.org/rsbl/article/11/7/20150480/50718/Changes-in-the-background-losses-of-woody-plant
https://www.science.org/doi/10.1126/science.aax9931
https://www.nature.com/articles/s41586-023-06861-4
But see: https://onlinelibrary.wiley.com/doi/10.1111/gcb.70587
Lines 164-165) This last part doesn't make sense to me, why would this provide a less informative signal?
Lines 165-169) Isn't one important aspect missed here the relative timing of herbivory in the season, or the proportion of annual herbivory at a site which is concentrated in the spring. There will likely be substantial variation in this aspect with important implications for budburst, sites/tree which have much more herbivory in the spring should benefit much more from delayed budburst than sites/trees where herbivory is more evenly distributed through the summer, or that occurs later.
Line 169) This expectation needs to be clearly described earlier in the introduction, preferably explicitly linked to one of the 3 study questions. I'm not sure why you would expect this, wouldn't you expect that the more severely attacked sites, there would be relatively greater benefit in delaying budburst.
Figure 2) "Weakest" is currently misspelled. Some more clarification in the text and/or caption is needed about what you mean by the strongest and weakest site-years.
Lines 196) Related how? Always give specific magnitude/direction of relationship if possible/appropriate, this is much more informative
Line 200) "before-year" isn't really used though it makes good sense, more usual would be to say "prior/previous year"
Lines 200-201) Not clear what you mean by "between-herbivore competition" which of the two factors mentioned does this apply to, or does it apply to both?
Lines 208-209) This is a remarkable result. Here you give some estimate of the mean/range in delays encountered. Given that the delays quite short (just a few days), this suggests that in this system/moth a huge portion of the annual herbivory occurs in just a few days.
Lines 222-223) This makes it sound like somehow delayed budburst causes more rapid maturation, whereas I guess they are just associated together. Plants that delay budburst are under greater pressure to "catchup". If delaying budburst is such a profitable strategy, and if any delays can be compensated for by more rapid development…why don't all trees use this strategy?
Lines 227-228) This expectation needs to be better explained/justified in the introduction.
Lines 230-232) Again, this expectation was not clearly described/motivated earlier in the introduction. Also, can you describe in words what this interaction means, to help with interpretation.
Lines 234-235) Its not clear to me why this result challenges this assumption. The interaction you observed doesn't mean that trees in the outbreak weren't "overwhelmed" whatever you mean by this, it just means that their strategy the next year was different. Am I misunderstanding something?
Line 240) This should be "predicting", also you can remove the "a" at the end of this line.
Lines 256-258) Fascinating result. But I'm a bit confused, if you're interested in the magnitude of the response (of budburst and herbivory reduction) shouldn't you use the slope of the relationships, not their r2? The r2 of a strong relationship (greater response of Y to a change in X) can still be lower than a relatively weak relationship. Similarly, you can easily of course have two sites with the same relationship between Y and X but very different r2 values. Though generally there will tend to be a positive correlation between slope and r2
Lines 293-296) Very interesting discussion. Somewhere perhaps here you need to acknowledge the extent to which your conclusions may be limited by the particularities of the herbivore species studied. Also, are there any rough estimates of what portion of the herbivore communities have narrow vs broad temporal niches. For this to have the major, broadly applicable implications you are seeking to make there needs to be a sizeable component of many herbivore communities which have a narrow temporal niche….is this the case?
Line 311) Should be "complement to the".
Line 313) I suggest you delete "major", it's quite vague, subjective in this case.
Lines 314) Effective yes, widespread is a stretch I think. You demonstrate this over a large area but still within oak forests in central Europe. Some extra discussion on differences/similarities between your study system (including herbivore

communities) and others, would help the reader to judge how widespread these results are likely to be.
Lines 317-322) OK, now you make this case, good. This should come already in the introduction, preferably with references supporting your claims.
Lines 331) You never really clearly made the point in the introduction that anthropogenic activity might have an effect of the spatial and temporal pattern of herbivory.

Version 1:

Reviewer comments:

Reviewer #1

(Remarks to the Author)
The authors satisfactorily addressed most of my critiques on the previous version. They tested the possible confounding effect of tree growth, drought and provided important references (with experimental evidence) supporting the significant 55% herbivory reduction following only 3 days delay in budburst. They clarified the accuracy of their methods. The study and findings appear more convincing now. I have only more minor remarks.

*L49: “often fails”: be more accurate: mention that the advancing of spring budburst slowed down though spring warming continues (Fu et al 2016 Nature)
*L74-75 vs L76-78: you present two cases and your hypothesis follow the first case; why this choice? Why not the second? There is no argumentation.
*L79: you are not working “on a tree” level (but pixel of canopy)
*L107: Hypothesis 4: you mean even in case the delay (in days) would be the same?
*Figure 1: why do you differentiate between spring and summer herbivory? This topic was only slightly touched in the “Discussion” without any definition … It is confusing; why not only mention “herbivory”? Also, to make it clear, you could add another schematic tree on the left for each case: above, starting with a tree under attack of few caterpillars (as in year t+1) and below, starting with a trees under severe attack of many caterpillars ….
*L163-167: you give quite high emphasis to your “first evidence” of a phenological mosaic but all field phenology observers are aware of inter-trees and spatial variability (see e.g. papers Nicolas Delpierre). I would simply give less emphasis (you described spatial variability nicely here but we know it since long time).
*Figure 2 is still difficult to follow (without reading the long legend), while the figure should be ideally easily to follow and to show after publication (e.g. in presentations, textbooks etc). First, I would add t and t+1 to clarify. Second, represent in a visual way “early/later budburst” or “greater herbivory” for axes (indices are rather unknown to many phenologists). Third, why you give so much important to the comparison to sites with highest and lowest R2? I would present this in Supplementary. Instead, I suggest to present here a gaussian with distribution of slopes for your 240 site-year combination (to give idea that large majority has positive or negative slope) and indicate the % of these cases that are significant. Something visually similar to Figure 2 in www.pnas.org/cgi/doi/10.1073/pnas.1321727111 (though there authors looked something completely different) ….. Also I would really not present in Extended Material the (super boring) two tables (they give the numbers but they are absolutely not visual and do not provide any synthesis ….)
*L235: “first evidence”? There were many experimental studies before. Formulate better ….
*L313: it is not the first time you mention “abiotic conditions (as inferred from indicator … of herbaceous vegetation)”. Have you described this in details? Where if not in Methods?

Reviewer #2

(Remarks to the Author)
Most of my comments were about clarity in the text of the manuscript. The authors have addressed my comments well, providing substantial new, relevant detail particularly on the study questions and motivations, and on the methods. I believe the resulting revised manuscript gives a clear account of an important study in a field of wide ecological interest

Decision Letter:

25th February 2026

Dear Dr. Mallick,

Thank you for submitting your revised manuscript "Satellite data show trees delay budburst across landscapes to escape herbivores" (NATECOLEVOL-25114016A). On the basis of the reviewers' comments (provided below), we will be happy in principle to publish it in Nature Ecology & Evolution, pending minor revisions to satisfy the reviewers' final requests and to comply with some editorial requests and our formatting guidelines.

We are now performing detailed checks on your paper and will send you a checklist detailing our editorial and formatting requirements in about two weeks (normally this takes less time but I will be travelling next week). Please do not upload the final materials and make any revisions until you receive this additional information from us.

Thank you again for your interest in Nature Ecology & Evolution. Please do not hesitate to contact me if you have any questions.

Best wishes,
[Redacted]

Reviewer #1 (Remarks to the Author):

The authors satisfactorily addressed most of my critiques on the previous version. They tested the possible confounding effect of tree growth, drought and provided important references (with experimental evidence) supporting the significant 55% herbivory reduction following only 3 days delay in budburst. They clarified the accuracy of their methods. The study and findings appear more convincing now. I have only more minor remarks.

*L49: “often fails”: be more accurate: mention that the advancing of spring budburst slowed down though spring warming continues (Fu et al 2016 Nature)
*L74-75 vs L76-78: you present two cases and your hypothesis follow the first case; why this choice? Why not the second? There is no argumentation.
*L79: you are not working “on a tree” level (but pixel of canopy)
*L107: Hypothesis 4: you mean even in case the delay (in days) would be the same?
*Figure 1: why do you differentiate between spring and summer herbivory? This topic was only slightly touched in the “Discussion” without any definition … It is confusing; why not only mention “herbivory”? Also, to make it clear, you could add another schematic tree on the left for each case: above, starting with a tree under attack of few caterpillars (as in year t+1) and below, starting with a trees under severe attack of many caterpillars ….
*L163-167: you give quite high emphasis to your “first evidence” of a phenological mosaic but all field phenology observers are aware of inter-trees and spatial variability (see e.g. papers Nicolas Delpierre). I would simply give less emphasis (you described spatial variability nicely here but we know it since long time).
*Figure 2 is still difficult to follow (without reading the long legend), while the figure should be ideally easily to follow and to show after publication (e.g. in presentations, textbooks etc). First, I would add t and t+1 to clarify. Second, represent in a visual way “early/later budburst” or “greater herbivory” for axes (indices are rather unknown to many phenologists). Third, why you give so much important to the comparison to sites with highest and lowest R2? I would present this in Supplementary. Instead, I suggest to present here a gaussian with distribution of slopes for your 240 site-year combination (to give idea that large majority has positive or negative slope) and indicate the % of these cases that are significant. Something visually similar to Figure 2 in www.pnas.org/cgi/doi/10.1073/pnas.1321727111 (though there authors looked something completely different) ….. Also I would really not present in Extended Material the (super boring) two tables (they give the numbers but they are absolutely not visual and do not provide any synthesis ….)
*L235: “first evidence”? There were many experimental studies before. Formulate better ….
*L313: it is not the first time you mention “abiotic conditions (as inferred from indicator … of herbaceous vegetation)”. Have you described this in details? Where if not in Methods?

Reviewer #2 (Remarks to the Author):

Most of my comments were about clarity in the text of the manuscript. The authors have addressed my comments well, providing substantial new, relevant detail particularly on the study questions and motivations, and on the methods. I believe the resulting revised manuscript gives a clear account of an important study in a field of wide ecological interest

Open Access This Peer Review File is licensed under a Creative Commons Attribution 4.0 International License, which permits use, sharing, adaptation, distribution and reproduction in any medium or format, as long as you give appropriate credit to the original author(s) and the source, provide a link to the Creative Commons license, and indicate if changes were made.
In cases where reviewers are anonymous, credit should be given to 'Anonymous Referee' and the source.
The images or other third party material in this Peer Review File are included in the article’s Creative Commons license, unless indicated otherwise in a credit line to the material. If material is not included in the article’s Creative Commons license and your intended use is not permitted by statutory regulation or exceeds the permitted use, you will need to obtain permission directly from the copyright holder.
To view a copy of this license, visit https://creativecommons.org/licenses/by/4.0/

Dear reviewers,

We sincerely thank you both for reviewing our manuscript, *"Satellite data show trees delay budburst across landscapes to escape herbivores".* We are grateful for your careful, constructive and thoughtful comments. Your feedback prompted substantial improvements in the clarity, rigor, and robustness of our study. We have carefully addressed all concerns and revised the manuscript accordingly. Below, we provide the point-by-point responses to each comment (responses in blue with revised text in *italics*). Here, we briefly summarize the major actions we have taken in response to your critiques:

1. **Accuracy and robustness of budburst and herbivory data (Reviewer 1).**
   We have substantially clarified and expanded the Methods and Introduction to:
   - explain the temporal resolution of the normalized canopy development index (NCDI), highlighting that single-day trajectories were used and validated against both Sentinel-2 and terrestrial laser scanning (TLS) data, ensuring that daily-scale changes are reliably captured.
   - clarify the spatial resolution of our study, stating that each 10 × 10 m Sentinel-1 pixel approximates the mean area of a mature tree crown and is suitable for canopy-level phenological inference, but does not represent one-to-one tree-pixel mapping. Previous wording implying exact tree correspondence has been corrected.
   - provide independent experimental validation of pixel-level herbivory estimates using a 2019 insecticide manipulation during a pest outbreak. Pixel-level herbivory was strongly suppressed in treated plots, confirming that the indices reflect actual herbivore activity.
   - discuss the potential for random noise in radar-based phenology estimates and explain why this is unlikely to have generated the consistent interannual ecological patterns we observed.
2. **Mechanistic basis of herbivory-induced budburst delay (Reviewer 1).**
   To address the possibility that delayed budburst could be a non-selected consequence of reduced tree resources, we incorporated independent measures of tree growth as a covariate in our analyses. Herbivory remained a highly significant predictor of budburst delay even after accounting for variation in growth rates, showing that resource depletion alone cannot explain the observed patterns. We thank the reviewer for suggesting this possibility and now discuss this alternative explanation in the discussion section.
3. **Abiotic confounding factors (Reviewer 1).**
   We repeated all analyses including abiotic indicator values (light, temperature, continentality, moisture, soil reaction, nutrient availability) based on plant community composition as covariates. Herbivory–budburst relationships did not change. Moreover, herbivory in the 2018 drought year did not differ from normal years, again ruling out drought as a confounding factor.
4. **Magnitude of herbivory reduction from a ~3-day delay (Reviewer 1).**
   We now include references to studies which have provided experimental evidence that a 3-day mismatch in the timing of egg hatching relative to budburst can reduce caterpillar survival to <10%, which is consistent with our observed ~55% reduction in herbivory. The studies we discuss provide mechanistic support for the strong protective effect of even small phenological shifts.
5. **Clarity of hypotheses and expectations (Reviewer 2).**
   The Introduction has been revised to clearly articulate the mechanisms behind our predictions for Questions 1 and 2, including why the responses are expected to weaken at

outbreak sites. These expectations are now numbered and referenced consistently throughout the manuscript.

6. **Statistical presentation of Fig. 3 (Reviewer 2).**
   We added analyses using slopes in addition to R^2, showing strong correlations between budburst-driven herbivory reduction and herbivory-driven budburst delay, further reinforcing our conclusions about adaptive plasticity.

7. **Improved clarity and transparency throughout the manuscript.**
   We refined our figure labelling, methodological descriptions, and discussion of limitations, ensuring that all key assumptions, potential sources of noise, and supporting experimental evidence are clearly presented.

Together, these revisions substantially strengthen the mechanistic plausibility, statistical robustness, and interpretative clarity of our findings. We believe the revised manuscript fully addresses the concerns raised and meets the standards for publication. We thank you again for your time and insightful feedback.

Sincerely,
Soumen Mallick on behalf of all co-authors

Reviewer 1:

In this study, the authors claim the finding that prior-year herbivory delays budburst of next year, and that such delay can substantially reduce herbivory damage (55%) in that next year. They suggest this to be an adaptive plastic response. The study was based on spatial-temporal analysis of radar data (Sentinel 1) for 60 oak forests (each of ca. 4.6 ha on average) in Southern Germany (some of which comprising ancillary data of insecticide application and/or herbivores natural antagonists). I must say I'm not fully convinced by the study, but I'm open to be corrected, if I misunderstood some points. My main reservations are listed below.

⇨ Thank you very much for your careful reading of our manuscript and for clearly articulating your reservations. You have raised some valid concerns, and we are grateful for the opportunity to clarify and reinforce the empirical and methodological basis of our study. In response to your comments, we have substantially expanded the explanation of our remote-sensing approach, added new analyses to rule out alternative explanations, incorporated additional experimental evidence, and revised several sections of the manuscript for clarity and transparency.

1. Before any inference and data analysis, these is the need to be sure about the accuracy of the budburst and defoliation data. The authors should explain (shortly, in main text) and in more details (in methods), the method of Bae at al 2022, which is at the foundation of their study (I find the details currently available too limited). To have information and insights on the methods, I had to go to the original paper of Bae at al 2022. So, I have realized that in the original Bae et al. study, (i) the analysis was done at plot level (4.6 ha) and not at the tree level, (ii) that the (very important) validation (with TLS data) was based on area of ca. 0.5 ha and that (iii) the index they developed (and fundamental in this study as shown in Extended Data Fig 3) is based on 12 days aggregated point. Based on this, you should convince the reader that your data at 'tree level' and detecting the short interval of '3 days' delay are robust, considering also all biases with radar assessment (also mentioned in Bae et al). My knowledge is limited, but I know few tree level RS assessments of phenology, one of which Eitel at al 2023 (https://doi.org/10.1016/j.agrformet.2023.109549), using much higher resolution data (PlanetScope, 3 m and 1 day). Also your statement that "27.500 10 x 10 pixels, each primarily corresponding to an individual tree crown" should be substantiated, as, for this statement, you would need tree density, location detected by radar and crown size, all matching, which personally I find it difficult. Overall, if there is uncertainty in the budburst/defoliation data and the index used (see Extended Data Fig 3), I would argue that only a combination of these data with a controlled manipulative experiment on herbivores could give a clear answer to your questions.

⇨ Thank you for raising this important point regarding the accuracy and resolution of the budburst and defoliation estimates and for prompting us to clarify both the temporal and spatial robustness of the satellite-derived indices. In response, we have substantially expanded the explanation of our remote-sensing approach in the Methods (lines 596-666, clean version) and added some brief additional description in the Introduction (lines 119-126, clean version). Specifically, we see that you had raised six points and respond to them one by one below. We note that the last point is probably the most important one.

(i) The analysis of Bae et al. being done at plot level (4.6 ha) and not at the tree level:

→ We now state in the manuscript: *"In that study[37], Sentinel-2 data were aggregated at the forest site scale (4.6 ha) …"* Most importantly, we contribute further analysis to ensure the correctness of the data as suggested. See our below response to point (vi).

(ii) The validation (with TLS data) of Bae et al. being based on area of ca. 0.5 ha.
→ We now state in the manuscript: "*In that study[37]… terrestrial laser scanning data were aggregated over 0.5 ha at site-centres..*".

(iii) The index Bae et al. developed being based on 12 days aggregated point:
→ We now clarify that in addition to their 12-day-timestep analysis, Bae et al. (2022) had also developed and validated their method at a resolution of single-day: "*Bae et al[37] developed both 12-day composite and single-day smoothed NCDI time series, and the single-day measures derived from multiple stages of the temporal trajectory were validated against optical (Sentinel-2) and terrestrial laser scanning (TLS) data (see Figure 2 of Bae et al[37]), supporting confidence in our single-day budburst index.*" In this context, we have also updated the exemplary illustration in Extended Data Fig. 3 and its corresponding legend to ensure that readers do not assume that our study was conducted at a temporal resolution of a 12-day-timestep.

(iv) The use of much higher resolution data such as Planetscope:
→ We now justify the interest of Sentinel 1: "*Note that Sentinel-1 is cloud independent, little prone to saturation contrary to both Sentinel-2 and Planetscope, and corrected for effects of canopy structure, background reflectance, and atmospheric conditions[37,67]. Nonetheless, it may still contain errors that are not systematic biases capable of generating patterns, but rather random noise that can obscure true patterns. Despite this noise, the emergence of patterns consistent with ecological hypotheses strengthens our confidence in their relevance.*"

(v) The issue with statement that "27.500 10 x 10 pixels, each primarily corresponding to an individual tree crown":
→ To avoid misunderstanding, we now clarify that each pixel simply approximates the area of an average mature tree crown: "*These indices reliably capture canopy phenology and defoliation at the 10 × 10 m pixel scale, where each pixel approximately represents the size of an average tree crown. We analysed 27,500 pixels across 60 oak-dominated forest sites spanning 2,400 km² in southern Germany (Extended Data Fig. 1).*"

(vi) The need for a combination of remote sensing data with a controlled manipulative experiment on herbivores:
→ We now added new analyses to the Methods section: *"In the present study, we added a finer, pixel-level evaluation, again confirming the reliability of the herbivory index. For this purpose, we studied pixels from the outbreak year (2019) that had high herbivore densities as inferred from high numbers of egg masses measured in the field. We tested whether, independent of site identity, the pixels that were treated by aerial insecticide application had lower herbivory index values than pixels that had not been treated. We confirmed that this was the case (Extended Data Fig. 2, LMM $z = 18.422$, $p < 0.001$). Furthermore, in the*

preceding years when no insecticide was applied, the same two groups of pixels did not differ (z = -0.336 and -0.486, both p ≈ 1).”

2. The most accurate projections that we can have today about budburst timing are based on (though incomplete) understanding and simulation of bud dormancy (see e.g. review 10.3389/fphgy.2023.1293057). How do you see your hypothesized conclusions (herbivory delaying next year budburst), fitting into this in term of processes and mechanisms? Would it not be, more simply, possible that the delay you see after herbivory and defoliation, is a physiological response only triggered by a weakened tree status (by herbivory) on next year phenology e.g. lower nutrients and C reserves? (see the advance in budburst related to nutrient status as described by doi.org/10.3389/fpls.2016.01641 and doi:10.1093/jxb/ert061, implicitly indicating later budburst in case of lower nutrients). Thus an interannual physiological response (legacy effect) rather than an adaptation?...

⇨ We have now conducted additional analyses accounting for resource level inferred from tree growth: “*Across five years and 60 forest sites, we tested whether leaf herbivory in year t predicted changes in the budburst ranks of pixels from year t to t+1. In support of Hypothesis 1, we found that higher herbivory in year t was related to later budburst in year t+1 (n = 110,000, F = 596.5, p < 0.001, adjusted R^2 = 0.102; Fig. 2a). This pixel-level pattern persisted even after accounting for potential effects of tree growth (Supplementary Method 3), indicating that the pattern is unlikely to result simply from herbivory depleting tree resources.” ...*
The pixel-level analyses given above were also conducted at the site level: *“These site-level patterns of adjusted R^2 remained robust (in every case p < 0.001, t = 4.513-11.951) even after controlling for potential effects of either tree growth, herbivore regulation by parasitoids or viruses, or abiotic conditions (as inferred from indicator values of herbaceous vegetation). Furthermore, the analyses had a higher adjusted R^2 (0.548) when none of these confounding factors were included than when either of the possible confounders were accounted for (0.347 to 0.480). Therefore, the delay of budburst with herbivory across dozens of tree populations suggests that budburst delay is not only a physiological response by individual trees to prior-year herbivory, or of natural enemies, but an adaptation selected in those tree populations where such a delay increases fitness most[35,36,43,56,57].”*

Please see the newly made Supplementary Methods 3 for the details of pixel-level analyses that account for tree growth, along with the corresponding results.

…In this context, had drought (e.g. in 2018) any impact on defoliation? Can you exclude any confounding effect with abiotic factors? (as you accurately have done for biotic factors in Supplementary Methods 2).

⇨ We have now conducted additional analyses accounting for abiotic factors and this is now mentioned in the main text: “*Using the same 5-year, 60-site dataset, we next tested whether changes in relative budburst timing from year t to t+1 of satellite pixels predicted changes in leaf herbivory over the same interval. In support of Hypothesis 2, herbivory decreased as budburst timing was delayed (n = 110,000, F = 1593.2, p < 0.001, adjusted R^2 = 0.149; Fig. 2e). This relationship remained robust after accounting for potential herbivore control by*

natural antagonists such as parasitoids and viruses, and abiotic factors as inferred from indicator values of ground vegetation (Supplementary Methods 2)." …
The pixel-level analyses given above were also conducted at the site level: *"These site-level patterns of adjusted R^2 remained robust (in every case $p < 0.001$, $t = 4.513$-11.951) even after controlling for potential effects of either tree growth, herbivore regulation by parasitoids or viruses, or abiotic conditions (as inferred from indicator values of herbaceous vegetation). Furthermore, the analyses had a higher adjusted R^2 (0.548) when none of these confounding factors were included than when either of the possible confounders were accounted for (0.347 to 0.480).*"

Please see the updated Supplementary Methods 2 for the details of analyses that account for abiotic factors, along with the corresponding results.

Regarding the 2018 drought specifically, we have now again conducted additional analyses and present them in Supplementary Methods *2*: *"In this context, we note that despite 2018 being the drought year in the region, it was not the year with highest herbivory. In 2019, the outbreak year, trees had significantly higher herbivory than in 2018 ($t = -11.503$, $p < 0.001$). Moreover, herbivory level in 2018 did not differ from 2020, a regular year without any particularity such as drought or herbivore outbreak ($t= -1.246$, $p = 0.598$)."*

3. In figure Extended Data Fig 3, it is clear that the herbivory period in the region in quite wide (> 60 days). Can you provide additional substantiation that only a 3 days shift would be so crucial in reducing so much herbivory damage (55%!)? Any experimental evidence showing similar results?

- ⇨ We now explain in the main text "*The average magnitude of herbivory reduction was remarkable. On average, pixels that delayed budburst profited from a 55% reduction in herbivory compared to the previous year (Supplementary Methods 1). Given the broad seasonal window of herbivore activity in the region (~60 days; Extended Data Fig. 3), such a substantial reduction resulting from only a three-day delay in budburst may seem surprising at first. However, a short delay can substantially increase herbivore mortality among the first larval instars, which are particularly vulnerable. This is supported by experimental studies showing that herbivores hatching just 3 days earlier than budburst exhibited mortality rates up to 90%*[47,48] …"

4. A more general comment: assuming that your conclusions are correct, should we not expect also a shift (delay) in phenology of herbivores too, following the delays of tree budburst? (in comparison than trees, their lifetime is so short and new generation so fast, that we can expect to be more adaptive in the long-term…..)

- ⇨ We appreciate this insightful comment. We now discuss the limited potential for herbivore adaptation to budburst delays of host trees: "*Importantly, because these budburst delays are transient and contingent on prior-year herbivory, they offer little opportunity for herbivores to adaptively shift their own phenology. Moreover, herbivore developmental timing is strongly constrained by climatic cues, which, under ongoing warming, generally favour earlier rather than later emergence. As a result, the intermittent and reversible budburst delays documented here are unlikely to drive long-term adaptive shifts in herbivore phenology*"

Reviewer 2:

This study examines the interactive effects and underlying drivers of budburst timing on herbivory in central European deciduous forests. The studies combines very impressive experimental manipulations across a large network of forests, with a novel series of remote sensing analyses. The results are highly novel and very convincing for the most part, they are highly relevant to anyone interested in understanding plant dynamics. The manuscript is generally well written though there are multiple, fairly minor areas which are rather unclear, and the analyses seem appropriate for the most part. I have two larger issues:

⇨ Thank you for your positive evaluation of our study and for the thoughtful comments. We appreciate the opportunity to further clarify the logic of our hypotheses and strengthen the interpretation of our analyses. We believe that the revisions we made in response to your comments have substantially improved the clarity, coherence, and impact of the manuscript. Below we address each of your points in detail.

(1) The logic and context underlying the study hypotheses/expectations need to be more clearly outlined in the introduction. This is particularly the case for questions 1 and 2, in particular the rationale for the expectations for why the responses in each case should be graded with a weaker response at outbreak sites. Then the results need to be more clearly tied to these questions. At several points in the results & discussion there are mentions of expectations and hypotheses and it's not clear if/how these overlap with the questions, and if so which specific question.

⇨ We have now substantially expanded the Introduction to explain why both the herbivory-induced budburst delay and the protective effect of that delay would be expected to weaken at outbreak sites. We have also named our 5 hypotheses, linked them with our 3 questions, and referenced the hypotheses consistently throughout the manuscript.

"Our Hypothesis 1 is thus that even across entire landscapes intense leaf herbivory on a tree in one year delays budburst in the following year." …

… "Therefore, a delay in budburst would lead to spring herbivores emerging before leaves are available (Fig. 1a), enabling trees to escape peak leaf herbivory by disrupting synchrony with their attackers (Hypothesis 2)."…

… "Because outbreak severity is driven largely by transient surges of herbivore populations across entire landscapes rather than by consistent or tree-specific factors[25–28], herbivory in an outbreak year is likely to be a poor predictor of herbivory risk for individual trees in subsequent years. Under these conditions, we expect that the herbivory-induced budburst delay may be lower at outbreak sites than elsewhere (Hypothesis 3). This effect of outbreak may be particularly seen if trees perceive outbreaks through increased light penetration[29] or through chemically mediated signals exchanged among conspecifics[30], mechanisms that enable trees to detect elevated herbivore pressure beyond their own direct experience. Moreover, the common assumption is that severe herbivore outbreaks overwhelm tree defences[26–28,31], such that even trees that delay budburst may still experience severe defoliation under extreme herbivore densities[21,31,32]. High herbivore densities also increase the likelihood of spillover from neighbouring trees[33], further reducing the effectiveness of

delayed budburst. Consequently, we expect that the herbivory reduction achieved through budburst delay may be lower at outbreak sites than elsewhere (Hypothesis 4)." …

… "Consequently, if budburst delay functions as a selected, adaptive defence, its magnitude should be greatest in forests where delaying budburst most reliably increases fitness by lessening herbivore damage (Hypothesis 5)."…

… "we addressed three key research questions:
1. Do trees delay budburst in response to prior-year herbivory (Hypothesis 1), and is this delay weaker at outbreak sites (Hypothesis 3)?
2. Does such a delay reduce subsequent herbivory (Hypothesis 2), and is this reduction weaker at outbreak sites (Hypothesis 4)?
3. Is the herbivory-driven budburst delay greatest in forests where it most efficiently reduces herbivory (Hypothesis 5)?" …

… "In support of Hypothesis 1, we found that higher herbivory in year t was related to later budburst in year t+1 (n = 110,000, F = 596.5, p < 0.001, adjusted R^2 = 0.102; Fig. 2a)."…

… "In support of Hypothesis 2, herbivory decreased as budburst timing was delayed (n = 110,000, F = 1593.2, p < 0.001, adjusted R^2 = 0.149; Fig. 2e)." …

… "We expected a weakened phenological response in outbreak sites, assuming that outbreak herbivory would provide a less reliable signal about risk of future herbivory (Hypothesis 3)[21,32]. … Contrary to our expectation (Hypothesis 3), the response was stronger at outbreak sites (n = 27,500, t = -3.588, p < 0.001; Fig. 2d)." …

… "We then assessed whether the relationship between budburst delay and herbivory reduction weakened in outbreak sites (Hypothesis 4). We therefore tested for a negative interaction term between outbreak (affected vs. unaffected sites) and budburst delay affecting the reduction of herbivory from 2018 to 2019 (the outbreak year). Contrary to our expectation (Hypothesis 4), we found a positive interaction term, meaning the relationship was stronger in outbreak sites (n = 27,500, t = 5.768, p < 0.001; Fig. 2h)" …

… "Across 240 site–year cases, these two metrics were positively correlated (df = 235, t = 16.769, p < 0.001; Fig. 3a), supporting Hypothesis 5." …

Please note that these changes also resolve the issues raised in your minor comments linked to earlier lines 84-85, 114-115, 164-165, 169, 227-228, and 230-232.

(2) I'm not clear that the analysis underlying fig 3 acheives what the authors intend. To compare the magnitude of two responses (budburst delay in response to herbivory & herbivory suppression in response to budburst delay) surely you should correlate the slopes of these two relationships? The r2 will tend to be positively correlated to the slopes, but captures rather different information to what you want to address your question I think.

⇨ Thank you for raising this important point. We now additionally analysed the slopes: "*When we repeated the analysis using slopes, an alternative measure of relationship strength, we again found a strong correlation (df = 235, t = –14.219, p < 0.001; Fig. 3b). Note that, as expected, the slopes are opposite in sign: trees exhibiting the greatest budburst delay in response to prior herbivory (negative slopes) occurred in forests where delayed budburst most effectively reduced subsequent herbivory (positive slopes).*"

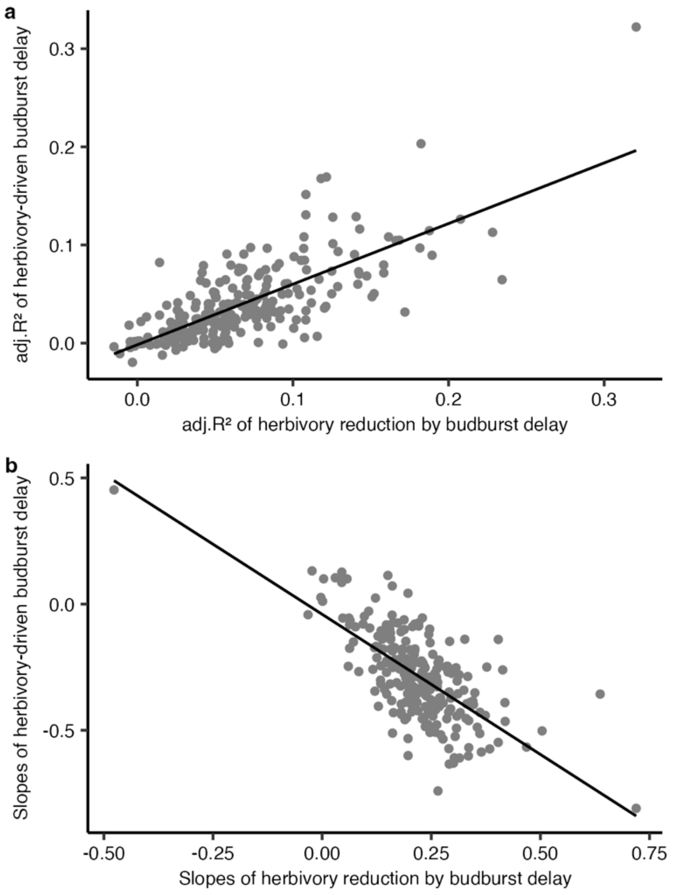

*"**Figure 3**: Coupling between the strength of herbivory-driven budburst delay and the strength of herbivory reduction by budburst delay, analysed across forest sites. The strengths of the relationships are interpreted from the adjusted R^2 values (**a**) and slope values (**b**) in Extended Data Table 1 and 2 (Note that relationships are expected to be positive in (a) and negative in (b)). The strength of herbivory-driven budburst delay increases with the strength of that delay in reducing subsequent herbivory, suggesting that trees delay budburst more strongly in forest sites where this strategy is more effective. This pattern helps explain why herbivory induces much greater delays in some forests than in others (Fig. 2b-c)."*

Minor comments:

Line 60) I like this observation. What is missing is some mention beforehand that herbivory itself may not be a static factor but may change directionally with climate change and other factors.

⇨ We now rewrote the beginning of the Abstract: "*Budburst, the timing of leaf emergence, often fails to advance as much as spring warming predicts, suggesting counteracting ecological forces. One of these forces might be increased and earlier herbivory on young leaves under climate warming. ...*"

Lines 56-58) This sentence felt like it should be the final conclusion.

⇨ We now placed this sentence at the end of the Abstract. The revised Abstract ends with "… *Ultimately, trees may be trapped between responding to two opposing consequences of global change: warming selects for earlier budburst, while herbivory selects for delay. Our results demonstrate the need to consider not only climate, but also plant-herbivore interactions and adaptive evolution to predict tree responses to a changing world.*"

Lines 65-66) Some brief review with relevant references would be useful here of other possible drivers of budburst, if there are any, besides climate change and herbivory.

⇨ Besides climate change and herbivory, photoperiod and nutrient reserves are other known major drivers of budburst penology. We now start the Introduction by briefly reviewing this topic with relevant references: *"Plant phenology has been studied in relation to numerous factors. Photoperiod[1,2], for instance, has been shown to prevent premature budburst (leaf emergence). In addition, the nutritional status of trees[3], particularly internal nutrient reserves accumulated in previous seasons[4], can influence phenological development of plants by affecting growth capacity and metabolic readiness. Plant phenology has probably been most extensively studied in relation to climate change[5–12], with one of the best-known examples being the earlier budburst of trees under elevated spring temperatures[6–10]. However, the advance in budburst under climate change has been slower than expected[12–14]. …*"

Lines 68-69) Rephrase "may erode tree responses", doesn't quite make sense at present and is rather vague, what responses, erode how/in what direction?

⇨ This sentence has become more specific in the revised manuscript: "*Such a herbivory-induced budburst delay may counteract the advancement of budburst under elevated spring temperatures.*".

Lines 74-75) Before this statement you should first clarify with evidence that spring is the peak time for herbivory. Also, can you give some assessment of what portion of the overall herbivore community are "spring-feeders".

⇨ We have restructured the paragraph following your suggestion. The revised paragraph reads: "*In temperate forests, the abundance of herbivorous insects typically peaks shortly after budburst, when young leaves are available and highly nutritious[17,21,22]. A substantial portion of the leaf-feeding insect community, often 60–80%, emerge as caterpillars during this period, making spring the peak time for herbivory[22–24]. Therefore, a delay in budburst would lead to spring herbivores emerging before leaves are available (Fig. 1a), enabling trees to escape peak leaf herbivory by disrupting synchrony with their attackers (Hypothesis*

2). This would result in herbivore mortality and a reduced overall herbivory[16,21]—a form of enemy escape in time that is rarely considered[16,25]. However, the evidence for such a mechanism is currently lacking."

Lines 84-85) This point is not clear to me, why does the fact that outbreaks last only a year mean that they are a poor proxy?

- ⇨ We apologize for our lack of clarity in the previous draft. We have improved the text, which now reads: "*Because outbreak severity is driven largely by transient surges of herbivore populations across entire landscapes rather than by consistent or tree-specific factors[25–28], herbivory in an outbreak year is likely to be a poor predictor of herbivory risk for individual trees in subsequent years. Under these conditions, we expect that the herbivory-induced budburst delay may be lower at outbreak sites than elsewhere (Hypothesis 3). This effect of outbreak may be particularly seen if trees perceive outbreaks through increased light penetration[29] or through chemically mediated signals exchanged among conspecifics[30], mechanisms that enable trees to detect elevated herbivore pressure beyond their own direct experience.*"

Lines 98-101) This first sentence of the paragraph seems unnecessary.

- ⇨ We removed this sentence and updated the next sentence (which is the current starting sentence) to ensure specificity.

Lines 114-115) I don't understand the last part of question 2, why would the response be weaker at outbreak sites? The background context and logic for the questions needs to be more clearly described in the introduction.

- ⇨ We have now more clearly developed hypothesis 3 and 4 in an earlier paragraph and refer to them here in the questions:

  … "Because outbreak severity is driven largely by transient surges of herbivore populations across entire landscapes rather than by consistent or tree-specific factors[25–28], herbivory in an outbreak year is likely to be a poor predictor of herbivory risk for individual trees in subsequent years. Under these conditions, we expect that the herbivory-induced budburst delay may be lower at outbreak sites than elsewhere (Hypothesis 3). This effect of outbreak may be particularly seen if trees perceive outbreaks through increased light penetration[29] or through chemically mediated signals exchanged among conspecifics[30], mechanisms that enable trees to detect elevated herbivore pressure beyond their own direct experience. Moreover, the common assumption is that severe herbivore outbreaks overwhelm tree defences[26–28,31], such that even trees that delay budburst may still experience severe defoliation under extreme herbivore densities[21,31,32]. High herbivore densities also increase the likelihood of spillover from neighbouring trees[33], further reducing the effectiveness of delayed budburst. Consequently, we expect that the herbivory reduction achieved through budburst delay may be lower at outbreak sites than elsewhere (Hypothesis 4)." …

  … "we addressed three key research questions:

1. Do trees delay budburst in response to prior-year herbivory (Hypothesis 1), and is this delay weaker at outbreak sites (Hypothesis 3)?
2. Does such a delay reduce subsequent herbivory (Hypothesis 2), and is this reduction weaker at outbreak sites (Hypothesis 4)?
3. Is the herbivory-driven budburst delay greatest in forests where it most efficiently reduces herbivory (Hypothesis 5)?" …

Lines 161-162) This is particularly important if there may be reasons to suggest that insect populations and herbivory could be consistently changing. See references below.
https://royalsocietypublishing.org/rsbl/article/11/7/20150480/50718/Changes-in-the-background-losses-of-woody-plant
https://www.science.org/doi/10.1126/science.aax9931
https://www.nature.com/articles/s41586-023-06861-4
But see: https://onlinelibrary.wiley.com/doi/10.1111/gcb.70587

⇨ Thank you. We have added this perspective in the sentence citing the literature that you provided. Now the sentence starts as "*It should be noted that most phenological models still project continued advancement of budburst under climate change[5,7,8,12,43], while rarely, if ever, accounting for biotic interactions such as herbivory—a major omission, given our findings and the fact that insect populations and herbivory seem to be consistently changing[44–46].*"

Lines 164-165) This last part doesn't make sense to me, why would this provide a less informative signal?

⇨ We now clearly develop hypothesis 3 in an earlier paragraph:

"Because outbreak severity is driven largely by transient surges of herbivore populations across entire landscapes rather than by consistent or tree-specific factors[25–28], herbivory in an outbreak year is likely to be a poor predictor of herbivory risk for individual trees in subsequent years. Under these conditions, we expect that the herbivory-induced budburst delay may be lower at outbreak sites than elsewhere (Hypothesis 3). This effect of outbreak may be particularly seen if trees perceive outbreaks through increased light penetration[29] or through chemically mediated signals exchanged among conspecifics[30], mechanisms that enable trees to detect elevated herbivore pressure beyond their own direct experience." …

And, here, in the concerned paragraph, we refer back to the Hypothesis 3.

… "We expected a weakened phenological response in outbreak sites, assuming that outbreak herbivory would provide a less reliable signal about risk of future herbivory (Hypothesis 3)[21,32]. … Contrary to our expectation (Hypothesis 3), the response was stronger at outbreak sites ($n = 27{,}500$, $t = -3.588$, $p < 0.001$; Fig. 2d)." …

We hope it is now easier to understand why the signal is less reliable during outbreaks.

Lines 165-169) Isn't one important aspect missed here the relative timing of herbivory in the season,

or the proportion of annual herbivory at a site which is concentrated in the spring. There will likely be substantial variation in this aspect with important implications for budburst, sites/tree which have much more herbivory in the spring should benefit much more from delayed budburst than sites/trees where herbivory is more evenly distributed through the summer, or that occurs later.

- ⇨ Following your minor comment linked to lines 74-75, we now introduce this topic in the Introduction. Moreover, in the Discussion we state: "*In temperate deciduous forests, herbivore communities are typically dominated by spring-feeding Lepidoptera whose first instars have narrow temporal windows of high vulnerability[11,17,21,39,52]. By contrast, another fraction of the herbivore community has broader temporal niches, is less constrained by short delays in budburst, and tends to contribute proportionally more to later-season herbivory[21,22]. Therefore, the among-site variation of the magnitude to which budburst delay reduces herbivory may reflect the composition and flexibility of the local herbivore community[16,21].*" However, testing whether sites which have higher herbivory in the spring benefit more from delayed budburst than sites where herbivory is more evenly distributed through the summer (or occurs later), would require continuous (time dependent) monitoring of herbivory across all seasons. Currently, we only measure the cumulative herbivory, and only across one season. Therefore, such an analysis is beyond the scope of our study. We now acknowledge this limitation in response to your minor comment linked to lines 293-296 (below).

Line 169) This expectation needs to be clearly described earlier in the introduction, preferably explicitly linked to one of the 3 study questions. I'm not sure why you would expect this, wouldn't you expect that the more severely attacked sites, there would be relatively greater benefit in delaying budburst.

- ⇨ In the revised manuscript, we now clearly develop hypothesis 3 in the Introduction, link it with question 1, and refer it here:

  "Because outbreak severity is driven largely by transient surges of herbivore populations across entire landscapes rather than by consistent or tree-specific factors[25–28], herbivory in an outbreak year is likely to be a poor predictor of herbivory risk for individual trees in subsequent years. Under these conditions, we expect that the herbivory-induced budburst delay may be lower at outbreak sites than elsewhere (Hypothesis 3). This effect of outbreak may be particularly seen if trees perceive outbreaks through increased light penetration[29] or through chemically mediated signals exchanged among conspecifics[30], mechanisms that enable trees to detect elevated herbivore pressure beyond their own direct experience." …

  … "we addressed three key research questions:
  1. Do trees delay budburst in response to prior-year herbivory (Hypothesis 1), and is this delay weaker at outbreak sites (Hypothesis 3)?"…

  … "We expected a weakened phenological response in outbreak sites, assuming that outbreak herbivory would provide a less reliable signal about risk of future herbivory (Hypothesis 3)[21,32]. … Contrary to our expectation (Hypothesis 3), the response was stronger at outbreak sites ($n = 27{,}500$, $t = -3.588$, $p < 0.001$; Fig. 2d)".

Figure 2) "Weakest" is currently misspelled. Some more clarification in the text and/or caption is needed about what you mean by the strongest and weakest site-years.

⇨ To make this clearer, we modified the figure legend and the titles of the figure panels.

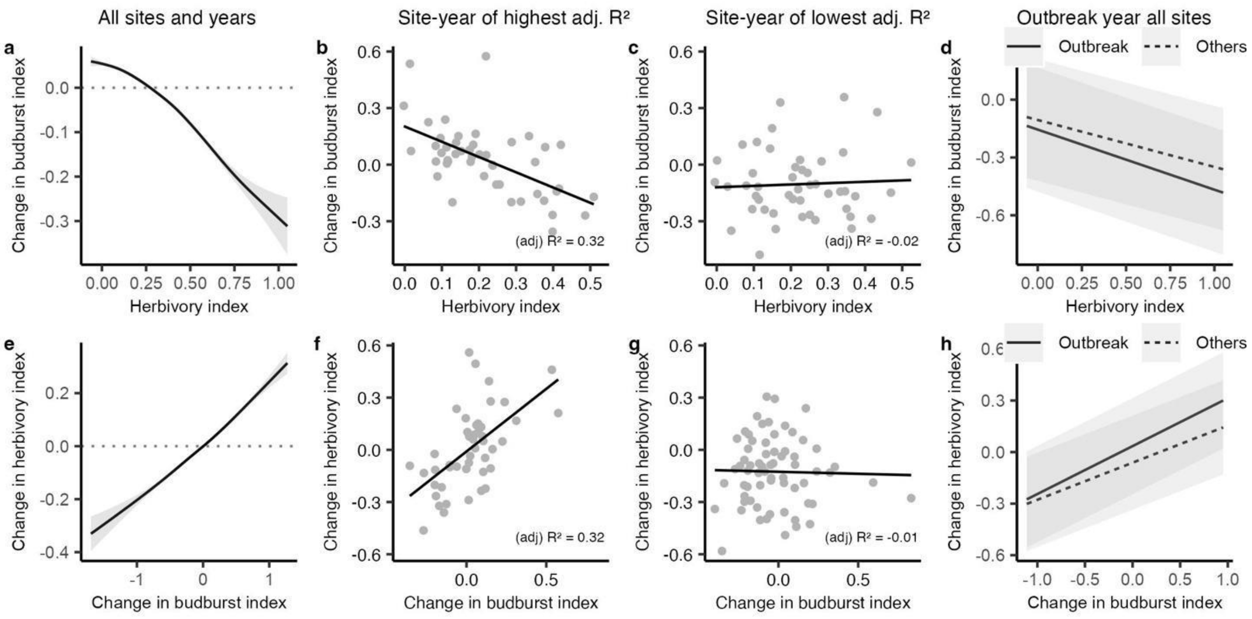

*"**Figure 2**: Observed relationships between budburst timing and leaf herbivory across forest sites and years. The top row (panels **a–d**) show the relationship between leaf herbivory in year t and changes in budburst index (i.e. ranking) from year t to t+1, and the bottom row (panels **e–h**) show how these changes in budburst timing predict changes in leaf herbivory over the same time interval. For **a–d**, higher x-axis values indicate greater leaf herbivory and positive y-axis values indicate earlier budburst; for **e–h**, positive x-axis values indicate earlier budburst and positive y-axis values indicate increased leaf herbivory. Across all sites and years, higher leaf herbivory was associated with delayed budburst the following year (**a**), though site-level relationships varied widely in adjusted R^2. Panels **b** and **c** illustrate the site–year combinations with the strongest and weakest adjusted R^2, respectively, and the relationship was somewhat more pronounced at sites experiencing spongy moth outbreaks (**d**). In turn, delayed budburst was associated with reduced leaf herbivory (**e**), again with strongest (**f**) and weakest (**g**) site-level relationships, and a somewhat more pronounced effect at outbreak sites (**h**). Solid lines are generalized additive model (GAM) predictions with shaded 95% confidence intervals, or with measured data as points. See Extended Data Tables 1–2 for site-specific results and Methods for model details."*

Lines 196) Related how? Always give specific magnitude/direction of relationship if possible/appropriate, this is much more informative

⇨ This sentence has been rephrased to "*In support of Hypothesis 2, herbivory decreased as budburst timing was delayed (n = 110,000, F = 1593.2, $p < 0.001$, adjusted $R^2 = 0.149$; Fig.*

2e)"

Line 200) "before-year" isn't really used though it makes good sense, more usual would be to say "prior/previous year"

⇨ Changes made in response to your subsequent comment resulted in the elimination of this formulation.

Lines 200-201) Not clear what you mean by "between-herbivore competition" which of the two factors mentioned does this apply to, or does it apply to both?

⇨ The text referenced here is not essential for interpretation of any of our main results. Therefore, to facilitate easier understanding, we decided to remove this less relevant part of the sentence.

Lines 208-209) This is a remarkable result. Here you give some estimate of the mean/range in delays encountered. Given that the delays quite short (just a few days), this suggests that in this system/moth a huge portion of the annual herbivory occurs in just a few days.

⇨ Thank you for appreciating the significance of this result (a similar point was also raised by reviewer 1 in her/his 3rd comment). We now discuss how a budburst delay of three days can possibly reduce herbivory by 55% even in a system with a long herbivory period: "*The average magnitude of herbivory reduction was remarkable. On average, pixels that delayed budburst profited from a 55% reduction in herbivory compared to the previous year (Supplementary Methods 1). Given the broad seasonal window of herbivore activity in the region (~60 days; Extended Data Fig. 3), such a substantial reduction resulting from only a three-day delay in budburst may seem surprising at first. However, a short delay can substantially increase herbivore mortality among the first larval instars, which are particularly vulnerable. This is supported by experimental studies showing that herbivores hatching just 3 days earlier than budburst exhibited mortality rates up to 90%*[47,48]."

Lines 222-223) This makes it sound like somehow delayed budburst causes more rapid maturation, whereas I guess they are just associated together. Plants that delay budburst are under greater pressure to "catchup". If delaying budburst is such a profitable strategy, and if any delays can be compensated for by more rapid development…why don't all trees use this strategy?

⇨ We rephrased the sentence to avoid causality and added another sentence to discuss the cost of delayed budburst. "*Moreover, delayed budburst is often associated with accelerated leaf maturation*[17,22] *so that leaves quickly enhance structural defences and chemical resistance, which further diminishes herbivore success.*" … "*Trees appear to choose to adjust their phenology based on recent herbivory, hence balancing*[34–36] *the benefits of herbivore escape against the possible costs associated with a shortened growing season*[43,58]*. Because such costs may reduce competitive dominance, trees often avoid delaying budburst to mitigate these effects.*"

Lines 227-228) This expectation needs to be better explained/justified in the introduction.

⇨ We now clearly develop hypothesis 4 in the Introduction, link it with our question 2, and refer to it here:

"Moreover, the common assumption is that severe herbivore outbreaks overwhelm tree defences[26–28,31], such that even trees that delay budburst may still experience severe defoliation under extreme herbivore densities[21,31,32]. High herbivore densities also increase the likelihood of spillover from neighbouring trees[33], further reducing the effectiveness of delayed budburst. Consequently, we expect that the herbivory reduction achieved through budburst delay may be lower at outbreak sites than elsewhere (Hypothesis 4)." …

… "we addressed three key research questions:
1. Do trees delay budburst in response to prior-year herbivory (Hypothesis 1), and is this delay weaker at outbreak sites (Hypothesis 3)?
2. Does such a delay reduce subsequent herbivory (Hypothesis 2), and is this reduction weaker at outbreak sites (Hypothesis 4)?" …

… "We then assessed whether the relationship between budburst delay and herbivory reduction weakened in outbreak sites (Hypothesis 4). We therefore tested for a negative interaction term between outbreak (affected vs. unaffected sites) and budburst delay affecting the reduction of herbivory from 2018 to 2019 (the outbreak year). Contrary to our expectation (Hypothesis 4), we found a positive interaction term, meaning the relationship was stronger in outbreak sites ($n = 27{,}500$, $t = 5.768$, $p < 0.001$; Fig. 2h)"

Lines 230-232) Again, this expectation was not clearly described/motivated earlier in the introduction. Also, can you describe in words what this interaction means, to help with interpretation.

⇨ We now explicitly explain the meaning of the interaction term: *"We then assessed whether the relationship between budburst delay and herbivory reduction weakened in outbreak sites (Hypothesis 4). We therefore tested for a negative interaction term between outbreak (affected vs. unaffected sites) and budburst delay affecting the reduction of herbivory from 2018 to 2019 (the outbreak year). Contrary to our expectation (Hypothesis 4), we found a positive interaction term, meaning the relationship was stronger in outbreak sites ($n = 27{,}500$, $t = 5.768$, $p < 0.001$; Fig. 2h)*"

Lines 234-235) Its not clear to me why this result challenges this assumption. The interaction you observed doesn't mean that trees in the outbreak weren't "overwhelmed" whatever you mean by this, it just means that their strategy the next year was different. Am I misunderstanding something?

⇨ We now elaborate this assumption: "*The observation that trees from outbreak sites with delayed budburst experienced reduced herbivory challenges the common assumption that severe herbivore outbreaks overwhelm tree defenses[26–28,31]—a scenario in which even trees that delay budburst would be expected to suffer severe defoliation under extreme herbivore densities[21,31,32]."*

Line 240) This should be "predicting", also you can remove the "a" at the end of this line.

⇨ Corrected as proposed.

Lines 256-258) Fascinating result. But I'm a bit confused, if you're interested in the magnitude of the response (of budburst and herbivory reduction) shouldn't you use the slope of the relationships, not their r2? The r2 of a strong relationship (greater response of Y to a change in X) can still be lower than a relatively weak relationship. Similarly, you can easily of course have two sites with the same relationship between Y and X but very different r2 values. Though generally there will tend to be a positive correlation between slope and r2

- ⇨ The same issue was also covered in your 2nd major comment, to which we responded by conducting an additional analysis with the slopes as suggested.

Lines 293-296) Very interesting discussion. Somewhere perhaps here you need to acknowledge the extent to which your conclusions may be limited by the particularities of the herbivore species studied. Also, are there any rough estimates of what portion of the herbivore communities have narrow vs broad temporal niches. For this to have the major, broadly applicable implications you are seeking to make there needs to be a sizeable component of many herbivore communities which have a narrow temporal niche….is this the case?

- ⇨ Thank you very much for raising this point. We now discuss it in an earlier paragraph: "*By postponing leaf emergence, trees reduce early-season herbivores' access to young, nutrient-rich foliage*[17]*. This phenological mismatch can suppress herbivore performance by delaying development, reducing feeding efficiency, and increasing mortality*[11,15,21,31]*. Moreover, delayed budburst is often associated with accelerated leaf maturation*[17,22] *so that leaves quickly enhance structural defences and chemical resistance, which further diminishes herbivore success. However, the effects of budburst delay may vary among herbivore communities. In temperate deciduous forests, herbivore communities are typically dominated by spring-feeding Lepidoptera whose first instars have narrow temporal windows of high vulnerability*[11,17,21,39,52]*. By contrast, another fraction of the herbivore community has broader temporal niches, is less constrained by short delays in budburst, and tends to contribute proportionally more to later-season herbivory*[21,22]*. Therefore, the among-site variation of the magnitude to which budburst delay reduces herbivory may reflect the composition and flexibility of the local herbivore community*[16,21]*.*"

Line 311) Should be "complement to the".

- ⇨ Corrected. The sentence now reads as "*These results demonstrate a phenology-driven pathway to delayed density dependence, offering a realistic alternative or complement to the classical mechanisms based on natural antagonists and chemical defences.*"

Line 313) I suggest you delete "major", it's quite vague, subjective in this case.

- ⇨ We had written 'major' as the same magnitude of advancement as triggered by more than a decade of climate warming. However, we understand that from common sense, 3 days does not sound major. So, we removed this word. The sentence now reads as "*Our findings demonstrate that herbivory-induced delays in budburst constitute a widespread and effective defence strategy, even during extreme outbreaks of herbivores.*"

Lines 314) Effective yes, widespread is a stretch I think. You demonstrate this over a large area but still within oak forests in central Europe. Some extra discussion on differences/similarities between your study system (including herbivore communities) and others, would help the reader to judge how widespread these results are likely to be.

⇨ Please see the response to the next comment.

Lines 317-322) OK, now you make this case, good. This should come already in the introduction, preferably with references supporting your claims.

⇨ We now discuss this information both in the Introduction and Discussion:

"The ecological dynamics of this region, including seasonally timed herbivore pressure, host-specific interactions, and substantial intra-specific variation in spring phenology, are characteristic of temperate deciduous forests worldwide, as demonstrated by global syntheses of phenology–climate interactions[10,38]*, classical work on temperate forest herbivore–host dynamics*[17,22,39]*, and cross-continental analyses of phenological variation in dominant deciduous tree species*[40,41]*."* …
… "*In temperate deciduous forests, herbivore communities are typically dominated by spring-feeding Lepidoptera whose first instars have narrow temporal windows of high vulnerability*[11,17,21,39,52]*. By contrast, another fraction of the herbivore community has broader temporal niches, is less constrained by short delays in budburst, and tends to contribute proportionally more to later-season herbivory*[21,22]*. Therefore, the among-site variation of the magnitude to which budburst delay reduces herbivory may reflect the composition and flexibility of the local herbivore community*[16,21]*.*"

Lines 331) You never really clearly made the point in the introduction that anthropogenic activity might have an effect of the spatial and temporal pattern of herbivory.

⇨ We understand that the angle of 'anthropogenic activity' comes out of blue as we did not develop it in the Introduction. Therefore, we have removed this text from the Conclusion. The updated Conclusion ends: "… *Finally, our study highlights that trees are experiencing opposing selection pressures from climate warming and herbivory, which may place them at risk of being caught in an evolutionary trap. Hence, we suggest that we it is only possible to fully understand tree response to abiotic changes—such as why budburst phenology often lags behind climate warming—by also considering biotic interactions.*"

Dear Reviewers,

We sincerely thank you for the careful evaluation of our manuscript, “*Satellite data show trees delay budburst across landscapes to escape herbivores*.” Your thoughtful and constructive comments have been extremely helpful in improving the clarity of the study. We improved the presentation of the results, including revisions to Figures 1 and 2 to enhance clarity and accessibility, and, as suggested, we reduced unnecessary emphasis on claims of novelty. Below, we provide detailed, point by point responses to each comment (responses in blue, with revised text shown in *italics*).

These revisions strengthen the clarity and interpretation of our findings. We believe the revised manuscript addresses the concerns raised and meets the standards for publication.

We thank you again for your time and insightful feedback.

Sincerely,
Soumen Mallick on behalf of all co-authors

Reviewer 1:

The authors satisfactorily addressed most of my critiques on the previous version. They tested the possible confounding effect of tree growth, drought and provided important references (with experimental evidence) supporting the significant 55% herbivory reduction following only 3 days delay in budburst. They clarified the accuracy of their methods. The study and findings appear more convincing now. I have only more minor remarks.

- ⇨ Thank you very much for your encouraging feedback. We now carefully addressed the minor remarks below.

*L49: "often fails": be more accurate: mention that the advancing of spring budburst slowed down though spring warming continues (Fu et al 2016 Nature)

- ⇨ We updated the sentence accordingly: "*In recent years, budburst, the timing of leaf emergence, has advanced less than expected despite continued spring warming, suggesting counteracting ecological forces.*"

*L74-75 vs L76-78: you present two cases and your hypothesis follow the first case; why this choice? Why not the second? There is no argumentation.

- ⇨ We have revised the text to clarify our reasoning: "*Small-scale studies (on ~20 individual trees[18,19]) have suggested that intense leaf herbivory on a tree in one year delays budburst in the following year. However, cases of advanced budburst after intense leaf herbivory have also been documented[15,20]. It remains unclear which of these patterns on individual trees scale up to entire landscapes, where budburst advancement has unexpectedly slowed[12-14]. The expected budburst advancement under elevated spring temperatures could be suppressed by the herbivory-induced budburst delay. Our Hypothesis 1 is thus that even across entire landscapes intense leaf herbivory in one year delays budburst in the following year.*"

*L79: you are not working "on a tree" level (but pixel of canopy)

- ⇨ We have removed the phrase "on a tree" from the sentence: "*Our Hypothesis 1 is thus that even across entire landscapes intense leaf herbivory in one year delays budburst in the following year.*"

*L107: Hypothesis 4: you mean even in case the delay (in days) would be the same?

- ⇨ We now clarify it: "*Consequently, we expect that for a given magnitude of budburst delay, the resulting reduction in herbivory may be lower at outbreak sites than elsewhere (Hypothesis 4).*"

*Figure 1: why do you differentiate between spring and summer herbivory? This topic was only slightly touched in the "Discussion" without any definition … It is confusing; why not only mention "herbivory"? Also, to make it clear, you could add another schematic tree on the left for each case:

above, starting with a tree under attack of few caterpillars (as in year t+1) and below, starting with a trees under severe attack of many caterpillars ….

⇨ We updated the figure accordingly.

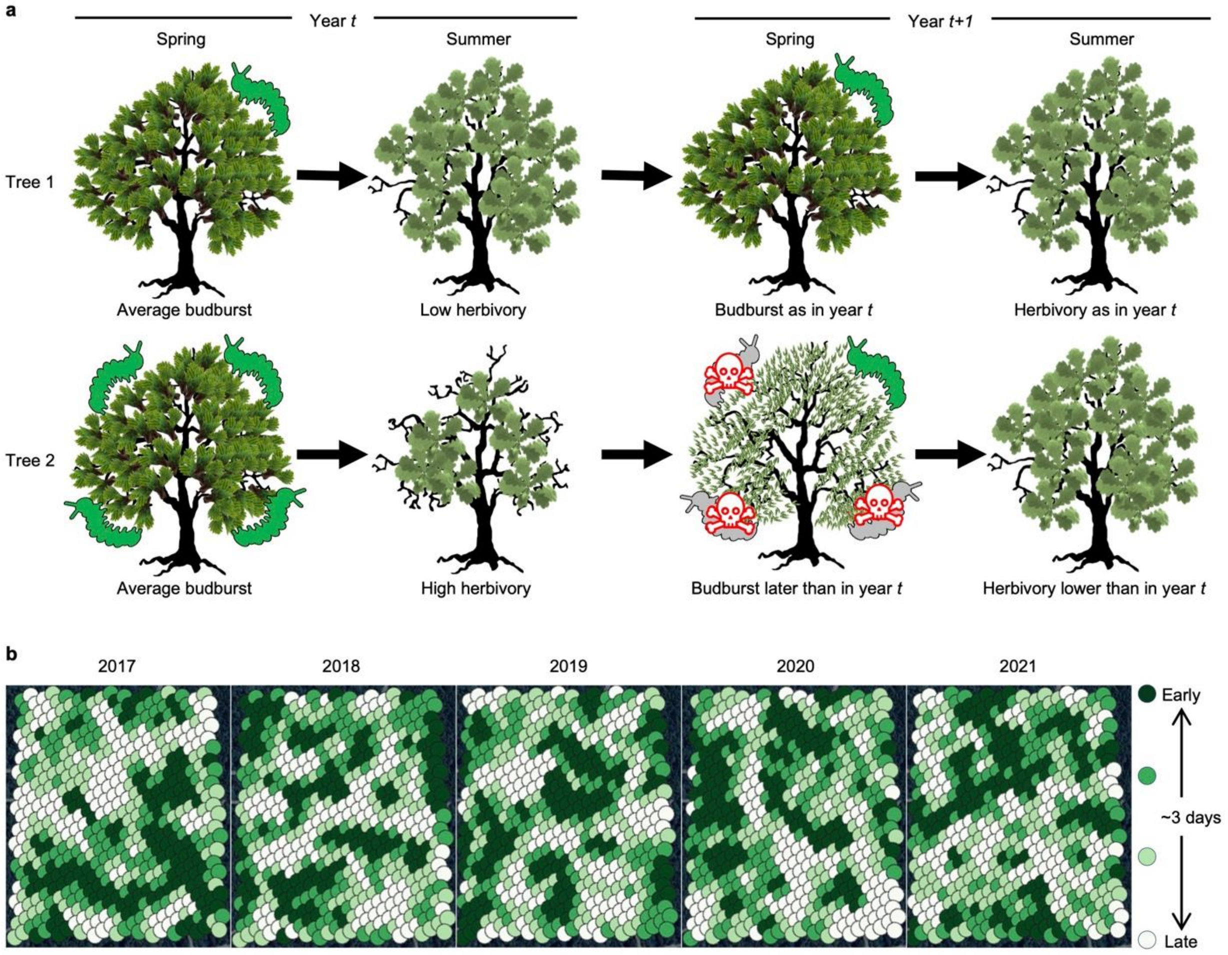

*L163-167: you give quite high emphasis to your “first evidence” of a phenological mosaic but all field phenology observers are aware of inter-trees and spatial variability (see e.g. papers Nicolas Delpierre). I would simply give less emphasis (you described spatial variability nicely here but we know it since long time).

⇨ We now give less emphasis: “*This reveals a ‘phenological mosaic’ across forests—a spatially and temporally structured pattern in the timing of bud development among individual trees, conceptually analogous to the mosaic cycle theory*[42] *for the development of entire trees. While spatial variation in phenology among tree individuals is well documented, our results suggest that it can organize into a cyclic spatiotemporal mosaic.*”

*Figure 2 is still difficult to follow (without reading the long legend), while the figure should be ideally easily to follow and to show after publication (e.g. in presentations, textbooks etc). First, I would add t and t+1 to clarify. Second, represent in a visual way “early/later budburst” or “greater herbivory” for axes (indices are rather unknown to many phenologists). Third, why you give so much important to the comparison to sites with highest and lowest R2? I would present this in Supplementary. Instead,

I suggest to present here a gaussian with distribution of slopes for your 240 site-year combination (to give idea that large majority has positive or negative slope) and indicate the % of these cases that are significant. Something visually similar to Figure 2 in www.pnas.org/cgi/doi/10.1073/pnas.1321727111 (though there authors looked something completely different) ….. Also I would really not present in Extended Material the (super boring) two tables (they give the numbers but they are absolutely not visual and do not provide any synthesis ….)

⇨ We updated the Figure 2, and removed the said tables from the Extended Data accordingly.

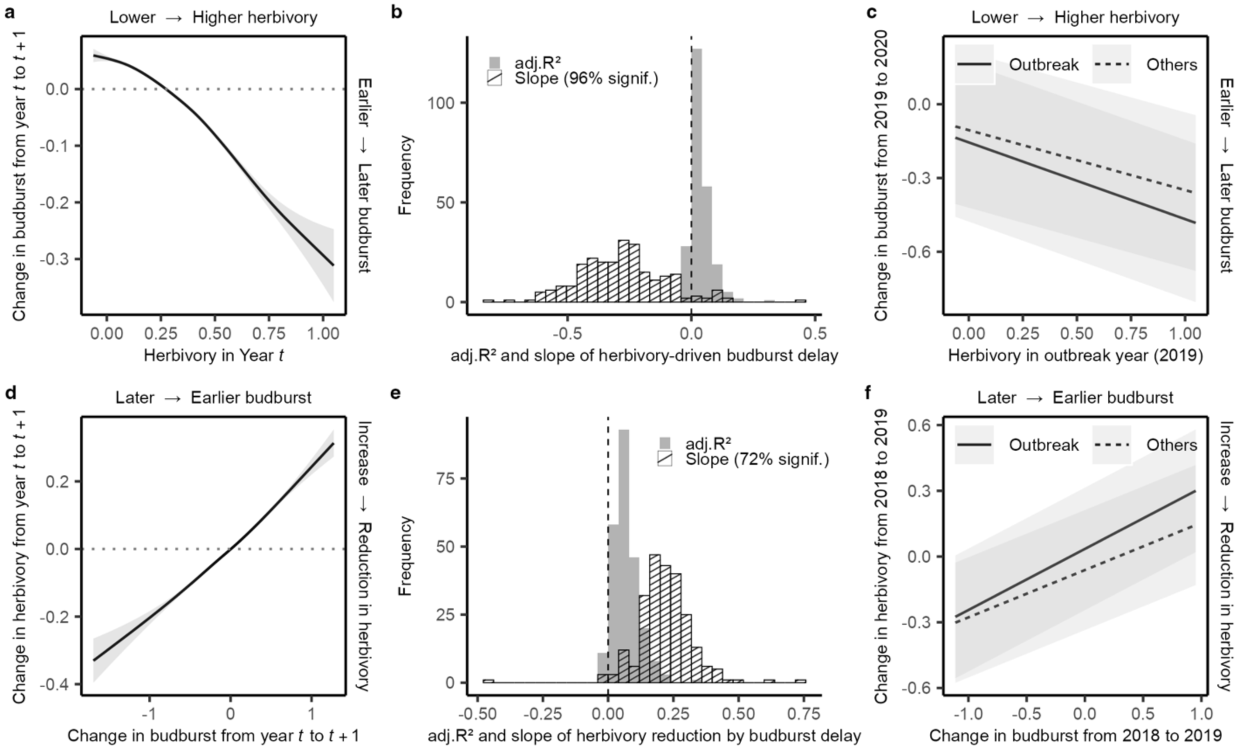

*L235: "first evidence"? There were many experimental studies before. Formulate better ….

⇨ The reformulated sentence reads: "*Thus, our results provide robust and large-scale support for the hypothesis that delayed budburst consistently reduces leaf herbivory by insects*".

*L313: it is not the first time you mention "abiotic conditions (as inferred from indicator … of herbaceous vegetation)". Have you described this in details? Where if not in Methods?

⇨ The detailed description is provided in Supplementary Methods 3, which was originally cited only at the first occurrence of this methodological description. Thank you for noting this point. We now also cite it here for clarity: "These site-level patterns of adjusted R2 remained robust (in every case $p < 0.001$, $t = 4.513$-11.951) even after controlling for potential effects of either tree growth, herbivore regulation by parasitoids or viruses, or abiotic conditions (as inferred from indicator values of herbaceous vegetation; see Supplementary Methods 3)"

Reviewer 2:

Most of my comments were about clarity in the text of the manuscript. The authors have addressed my comments well, providing substantial new, relevant detail particularly on the study questions and motivations, and on the methods. I believe the resulting revised manuscript gives a clear account of an important study in a field of wide ecological interest:

⇨ Thank you for your positive evaluation of our study.
